# Triazole and Pyrazole Hybrids of Electrophilic Natural Products as Promising Anticancer Agents

**DOI:** 10.3390/molecules31020355

**Published:** 2026-01-19

**Authors:** Alessia Da Fermo, Alessandra Bisi, Rebecca Orioli, Silvia Gobbi, Federica Belluti

**Affiliations:** Department of Pharmacy and Biotechnology, Alma Mater Studiorum—University of Bologna, Via Belmeloro, 6, 40126 Bologna, Italy; alessia.dafermo3@unibo.it (A.D.F.); alessandra.bisi@unibo.it (A.B.); rebecca.orioli3@unibo.it (R.O.); silvia.gobbi@unibo.it (S.G.)

**Keywords:** chalcone, curcumin, aurone, cancer, electrophilic natural products, hybrid, triazole, pyrazole

## Abstract

Naturally inspired electrophilic scaffolds, such as chalcone, curcumin, aurone, C-5-monocarbonyl-curcumin, and bis-(arylidene)piperidone, are considered privileged structures because of their ability to interact with a variety of biological macromolecules, including receptors and enzymes. They thus serve as versatile platforms for drug discovery efforts aimed at developing structurally related analogues endowed with improved bioactivity. Five-membered nitrogen-based heterocycles, such as triazole and pyrazole, have been widely used in medicinal chemistry both as templates and spacers for the design of bioactive compounds; they indeed provide the advantage of enhancing favourable interactions with the target, while also improving solubility and bioavailability, along with reducing toxicity. This review reports the latest advances in the development of hybrids incorporating the above classes of synthons acting as potential anticancer chemotherapeutics and provides a critical summary of the design strategies that have guided the development of antitumor agents.

## 1. Introduction

Currently, cancer remains a significant global public health issue in developed countries, being the second most common cause of death. According to the Global Cancer Observatory (GLOBOCAN) 19,976,499 [1] cases of malignancies were diagnosed worldwide, with lung, breast, colorectal, and prostate cancers being the most prevalent [2]. Available treatments such as chemotherapy, radiotherapy, hormone therapy, and immunotherapy are often associated with adverse effects. For example, the inability of anticancer agents to distinguish between cancerous and normal cells results in inherent toxicity, while the emergence of chemoresistance is associated with reduced clinical outcomes and long-term survival. In this scenario, cancer prevention and treatment represent significant public health challenges. Despite intensive research efforts, effective treatments remain elusive, emphasising the need to develop new, potent, and safe chemotherapeutics.

The molecular hybridisation strategy, by combining different pharmacophore units into a single chemical entity, has enabled the development of multitarget or multifunctional agents with broadened or improved biological activity, thus emerging as a promising medicinal chemistry approach [3,4,5,6]. Indeed, this approach also offers the promise of addressing several drawbacks by improving pharmacokinetic and pharmacodynamic properties, as well as bioavailability, thereby facilitating transport across cell membranes and providing protection from enzymatic breakdown.

Different design approaches to hybridisation can be categorised by how the underlying molecular frameworks, whether partial or complete structures, are assembled, resulting in conjugation, fusion, and merging as the main approaches.

(a) Conjugation by using a specific molecular linker that is chemically distinct from the connected synthons. A cleavable or non-cleavable tether can be used advantageously. In the first case, a metabolically unstable linkage facilitates the release of the unmodified bioactive synthons, which then interact independently with their respective biological counterparts. In the former, the main structures were covalently connected via the linker, ensuring the hybrid stability within the metabolic environment.
(b) Fusion of the selected pharmacophores or their components leads to their direct integration, with the parts coming into close contact.
(c) Merging of the selected pharmacophores, by exploiting the common chemical features among their structures, allows obtaining a smaller and simpler chemical entity [7].

Nitrogen-based heterocycles are regarded as “privileged fragments” in medicinal chemistry due to their metabolic stability and favourable binding properties, i.e., ability to make various non-covalent interactions, including hydrogen bonds, dipole–dipole interactions, and van der Waals contacts, with biological macromolecules. Among others, the five-membered rings triazoles and pyrazoles have been widely used in medicinal chemistry as pharmacophores, building blocks, and linkers [8,9,10,11,12,13], thus enabling the development of bioactive chemical entities across different therapeutic areas, including neurodegeneration, inflammation, and infectious diseases, with cancer being the most prevalent [14]. Moreover, they have proved to be stable molecular linkers, providing chemical stability, structural rigidity, and reliable connectivity to the designed hybrids. This peculiar versatility has been extensively exploited in drug discovery, strongly supporting the design and optimisation of bioactive compounds. In particular, 1,5- and 1,4-disubstituted 1,2,3-triazoles can be readily prepared by the Huisgen 1,3-dipolar cycloaddition, also known as Cu-catalysed azide-alkyne cycloaddition (CuAAC) or “click chemistry” reaction, which offers high yields, broad substrate suitability, and short reaction times [15].

Natural products (NPs) remain a limitless source of drug candidates and lead compounds for the design of new and effective bioactive chemical entities [16]. Several NPs belonging to the chalcones, curcuminoids, and aurones classes are characterised by a wide spectrum of bioactivities, including anti-inflammatory, antioxidant, and chemopreventive, which make them suitable for targeting the onset and progression of multifunctional disorders, such as cancers and neurodegeneration. This peculiar feature can arise from the presence of an electrophilic α,β-unsaturated carbonyl fragment in their structure, which can undergo thia-Michael reactions with cysteine-rich residues in biological macromolecules [17]. The versatility of these scaffolds has attracted interest in medicinal chemistry, where they are considered “privileged structures” for developing new therapeutics, as evidenced by many published papers.

The general classification of NPs as pan-assay interference compounds (PAINS) has sparked concerns about their druggability and has become a significant challenge in drug discovery. Actually, properly tailored modifications of NP-privileged scaffolds have enabled the development of bioactive agents able to selectively modulate well-defined targets. These outcomes thus demonstrate that the biological activity of NPs and their analogues can be deliberately exploited for intended purposes [18,19]. For instance, NPs functionalisation has successfully allowed the development of potential therapeutics acting through the modulation of different targets involved in tumour progression and development, namely histone deacetylases (HDACs), epidermal growth factor receptor (EGFR), vascular endothelial growth factor receptor (VEGFR), microtubules, topoisomerases, and nuclear factor-kB (NF-kB) [20].

## 2. Electrophilic Natural Product Hybrids as Anticancer Agents

**Figure 1:**
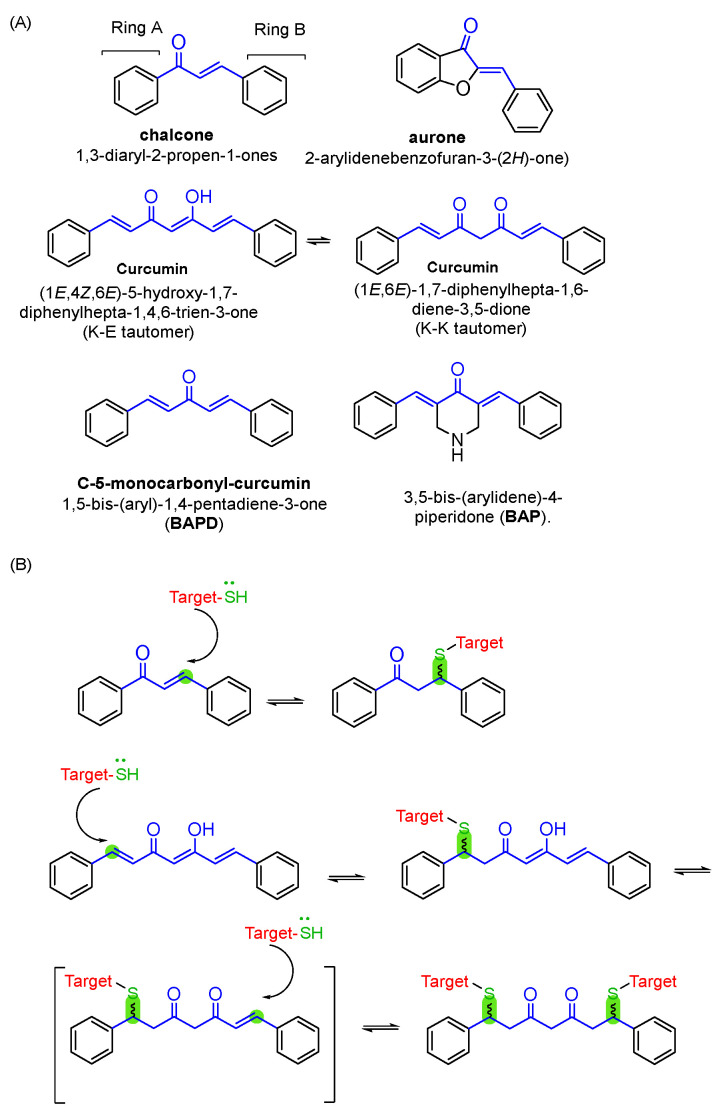
(**A**) Structure of the NPs-based electrophile scaffolds curcumin, chalcone, and aurone and the structurally modified templates BAPD and BAP (the α,β-unsaturated carbonyl fragment is highlighted in blue). (**B**) Michael Addition reaction of a thiol-based moiety to the electron-rich fragment of chalcone or curcumin main templates.

This review article will survey the most significant findings related to anti-cancer agents developed through the hybridisation strategy between an NPs-based electrophile scaffold, such as curcumin, chalcone, aurone, and the corresponding modified templates 1,5-bis(aryl)-1,4-pentadiene-3-one (BAPD) and 3,5-bis(arylidene)-4-piperidone (BAP) (Figure 1A), and a nitrogen-based heterocycle, particularly triazole and pyrazole.

Scaffolds containing an electrophilic α,β-unsaturated carbonyl group, known as a potential Michael acceptor, typically exhibit bioactivity. They are involved in regulating various signalling pathways and can be used as probes in chemical biology research [21]. The electrophilic moiety can readily undergo a Michael reaction, forming covalent bonds with the thiol groups of glutathione or cysteine residues, resulting in an adduct (Figure 1B) [22,23,24]. In biological systems, this process may be crucial for improving the bioactivity of molecules. For example, modulation of the Keap1-Nrf2-ARE pathway by covalent modification of Keap1 cysteine residues leads to the release of Nrf2, which in turn promotes the induction of phase II enzymes [17]. The electron density in the aromatic rings connected to the α,β-unsaturated carbonyl fragment influences its electrophilic character [25].

The present contribution reports on medicinal chemistry efforts involving various scaffolds bearing an α,β-unsaturated system. The aim is to provide a broad and comprehensive overview of N-heterocycle hybridisation-driven bioactivity in the context of electrophilic-based compounds. The top-performing derivatives among the chosen series of compounds were reported. In certain instances, an inactive representative compound was also included to underscore the chemical features crucial for inducing the anticancer effect. Indeed, comparing active and less active molecules provides valuable insights into how specific modifications may influence biological effectiveness. This enhances scientific understanding and supports the rational design of new bioactive compounds. Focus will be given to articles published between 2020 and 2025.

## 3. Chalcones

**Figure 2:**
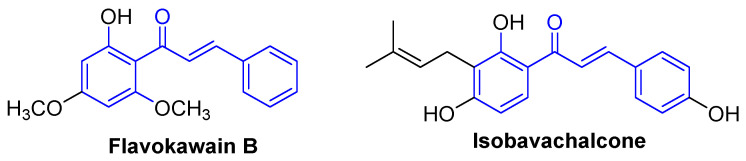
Structure of the naturally occurring chalcones Flavokawain B and Isobavachalcone [28,29].

Chalcones (1,3-diaryl-2-propen-1-ones; Figure 1A), members of the flavonoid family, play a prominent role among biologically active NPs due to their broad pharmacological profile, which includes anticancer, neuroprotective, anti-inflammatory, and antimicrobial effects [26]. Several natural chalcones are marketed or under clinical investigation for the treatment of various pathologies, including metochalcone (choleretic/diuretic) and sofalcone (antiulcer/mucoprotective) [27]. Flavokawain B and Isobavachalcone (Figure 2) were found to exhibit anticancer effects by inducing apoptosis, thereby increasing the Bax/Bcl-2 ratio or activating caspase-3 and -9; a blockage in the cell cycle at the G2/M phase has also been documented [28,29]. The chalcone scaffold has been extensively used in medicinal chemistry to develop effective anticancer agents [30,31]. Numerous structural analogues have been generated through appropriate functionalization of the aryl moieties, commonly referred to as Ring A and Ring B, which are adjacent to the carbonyl group and the C=C double bond, respectively (Figure 1A). Moreover, hybridisation of this scaffold through nitrogen heterocycles has been particularly successful, as evidenced by numerous primary studies and review articles [32,33].

### 3.1. Triazole-Based Chalcone Hybrids

**Figure 3:**
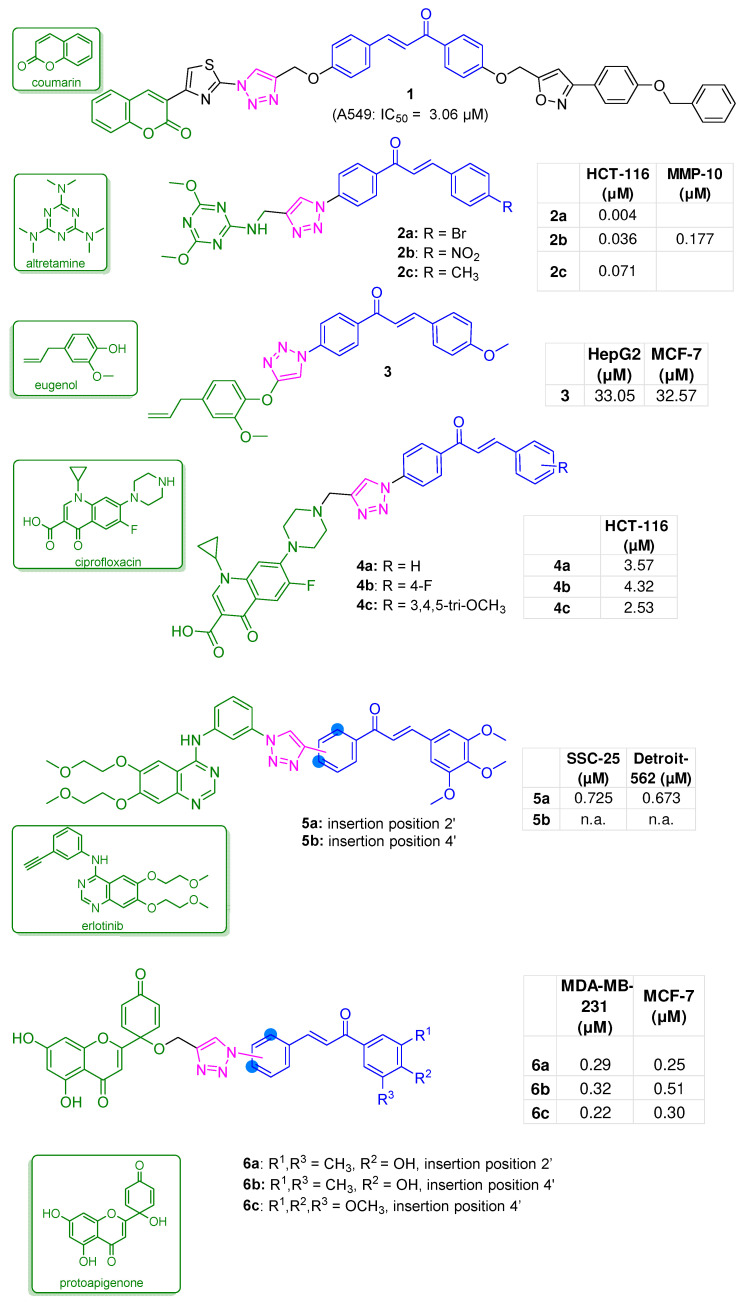
Structures of chalcone-1,2,3-triazole hybrids bearing antiproliferative pharmacophores (**1** [34], **2a**–**c** [38], **3** [40], **4a**–**c** [42], **5a**,**b** [43], **6a**–**c** [45]); bioactivities are reported in tables.

The hybrid **1** (Figure 3) was developed, characterised by a phenyl-benzyloxy-isoxazole function at the 4-position of the chalcone A-ring, in which a thiazole-coumarin moiety was inserted into the 4′-position of the B-ring through a 1,2,3-triazole linker. The compound exhibited good antiproliferative potency against A549 lung cancer cells (IC_50_ of 3.06 µM), while it showed a moderate effect on Capan-1 pancreatic cancer cells (IC_50_ of 65.41 µM). In contrast, when tested in the healthy L929 fibroblast cell line, **1** showed low cytotoxic effect (IC_50_ of 36.38 µM). In Capan-1 cells, the hybrid displayed a remarkable necrotic effect, likely due to its ability to induce nuclear condensation. Docking simulations on **1** revealed strong binding affinities to phosphodiesterase (PDE)-10A and the Epidermal Growth Factor Receptor (EGFR): −13.425 kcal/mol and −13.07 kcal/mol, respectively, indicating these as possible targets. Specifically, the following interaction patterns were noted: (a) PDE10A: H-bonds with Tyr683 and Gln716 (oxygen atoms of the coumarin moiety); van der Waals interactions with Met703, Ile701, Tyr720, Ser667, and Leu665; π–π stacking interactions with Phe686 (thiazole ring), and with Phe719 (coumarin ring). (b) EGFR: H-bonds with Lys745 (1,2,3-triazole ring); π–π stacking interactions with Phe723; π–cation interactions with Arg841, Lys745, and Arg803; and a water-bridged hydrogen bond involving the catalytic Mg^2+^ ion further stabilised the complex (Table S1) [34].

Matrix metalloproteinases (MMPs), zinc-dependent endopeptidases deregulated in nearly all human cancers, are regarded as validated anticancer targets [35]. In particular, inhibition of the MMP-10 and -13 isoforms has emerged as a strategy for managing colorectal cancer (CRC) [36]. Thus, a series of hybrid compounds was designed to obtain antitumor agents targeting MMPs. Considering the pharmacophore model for the design of MMP inhibitors, which encompasses an electrophilic pharmacophore linked through amido groups to a planar ring (Figure 3), the structures of these compounds included: (a) an aryl moiety, (b) an electrophilic warhead, (c) an amide bond, (d) a heteroaromatic function. Specifically, the chalcone scaffold (a–b) was connected via a triazole spacer (c), to an s-triazine moiety (d) found in the FDA-approved anticancer agent altretamine [37]. Among the designed compounds **2a**–**c** (Figure 3), potently inhibited colon carcinoma HCT-116 cell proliferation (IC_50s_ = 4.36 and 71 nM, respectively) and showed favourable selectivity indices, thus emerging as potential CRC therapeutic opportunities. Analogue **2b** was the only compound of the series that effectively inhibited MMP-10 (IC_50_ = 0.177 µM) without affecting MMP-13 [38].

A series of hybrids was created by connecting, via a 1,2,3-triazole spacer, the chalcone scaffold to eugenol (4-allyl-2-methoxyphenol), a prominent aromatic NP found in plants within the *Ocimum genus*, and endowed with an anticancer potential mainly related to pro-apoptotic effects [39]. Within the series, compound **3** (Figure 3) was the most effective, demonstrating a mild antiproliferative effect against the HepG2 human hepatocellular carcinoma and MCF-7 human breast cancer cells (IC_50s_ of 33.05 and 32.57 µM, respectively) [40].

To develop multipotent anticancer agents, a substituted chalcone scaffold, known for inhibiting tubulin polymerisation, was linked via a 1,2,3-triazole connector to the piperazine moiety of ciprofloxacin (CP). CP is a broad-spectrum antibacterial agent that has recently demonstrated anticancer properties by inhibiting topoisomerase-II, inducing apoptosis, and arresting the cell cycle [41]. Compounds **4a**–**c** (Figure 3) induced a remarkable antiproliferative effect in various cancer cell lines, proving particularly effective in colon HCT-116 cells (IC_50_ of 3.57, 4.32, 2.53 µM, respectively). In contrast, a very low toxicity profile was observed in normal human embryonic kidney (HEK) 293 cells. The effects on topoisomerase I and II inhibition were evaluated in HCT-116 cells. Notably, **4a**, with an unsubstituted chalcone, was the most potent in inhibiting topoisomerase I, followed by **4b** (4-F substituted), while **4c** (3,4,5-trimethoxy-substituted) was almost inactive. Concerning topoisomerase II inhibition, CP hybrids **4a**–**c** demonstrated notable effects even at a low concentration (10 µM). Docking studies on topoisomerase I (Top-I, PDB ID: 1T8I) showed that **4a** adopts a camptothecin-like binding mode, establishing three key H-bonds with Asp533, Lys532, and Arg364 (Table S1). In contrast, for topoisomerase II (Topo II, PDB ID: 6ZY7), **4a**–**c** displayed binding modes distinct from those of etoposide; in particular, **4a** formed H-bonds with Ala65 and Ser64, whereas **4c** interacted with Ser800 (Table S1). A significant disruptive effect on tubulin polymerisation was observed for **4c** (assayed at the IC_50_ value in HCT116 cells), thereby confirming the design strategy. DNA-damage studies in HCT116 cells revealed that, at their respective IC_50_ values, the above CP-chalcone hybrids induced DNA double-strand breaks, consistent with the interference in topoisomerase activity and microtubule dynamics. Moreover, **4c** was found to induce a G2/M phase cell cycle arrest [42].

Given the crucial role of EGFR in carcinogenesis, hybrid compound **5a** was designed by linking the EGFR inhibitor erlotinib to a 3,4,5-trimethoxy chalcone through a 1,2,3-triazole function (Figure 3). Analogue **5a**, in which the erlotinib pharmacophore is linked via a triazole linker to the chalcone A-ring at position 2′, demonstrated remarkable efficacy in inhibiting HNSCC cells viability (Detroit 562 cell line: IC_50_ = 0.673 µM, SSC-25 cell line: IC_50_ = 0.725 µM), higher than analogue **5b** (4′-insertion), erlotinib (Detroit 562 cell line: IC_50_ = 4035 µM, SSC-25 cell line: IC_50_ = 150 µM), and the unsubstituted 3,4,5-trimethoxychalcone (Detroit 562 cell line: IC_50_ = 3.074 µM, SSC-25 cell line: IC_50_ = 2.777 µM). Hybrid **5a**, at 2.5 µM, induced necrotic cell death (Detroit 562 cells) and triggered apoptosis (SCC-25 cells) by affecting mitochondrial membrane potential, cytoplasmic vacuolisation, and nuclear shrinkage. Unfortunately, unlike erlotinib, it did not inhibit EGFR, suggesting that an alternative mechanism underlies the observed antiproliferative effects [43].

The inhibition of ataxia-telangiectasia and Rad3-related protein (ATR)-mediated activation of checkpoint kinase 1 (Chk1), along with the induction of oxidative stress and suppression of mitochondrial membrane potential, is regarded as a valuable, multifunctional anticancer strategy. Modulation of these targets can enhance tumour sensitivity by inducing cell death or exploiting synthetic lethality [44]. In this context, hybrids were created by linking, via a triazole connector, the ATR inhibitor and oxidative stress inducer protoapigenone (Figure 3) [33] to the chalcone B-ring (at 2′- and 4′-positions). Compounds **6a**,**b**, with a 3,5-dimethyl-4-hydroxy substitution pattern, and **6c**, with a 3,4,5-trimethoxy substitution pattern, on the A-ring (Figure 3) demonstrated consistent cytotoxicity in cancer cell lines, specifically MCF-7 and MDA-MB-231 (sub-µM IC50 values), and outperformed cisplatin. Both **6a** and **6b** significantly induced late apoptosis, while **6c** altered the MDA-MB-231 cell cycle by increasing the hypodiploid (subG1) phase and decreasing the G1 phase, and triggered apoptotic cell death by increasing caspase-3 activity. Compound **6b** inhibited ATR-mediated Chk1 activation and induced mitochondrial membrane depolarisation, leading to disruption in the redox balance, thus validating the design strategy [45].

**Figure 4:**
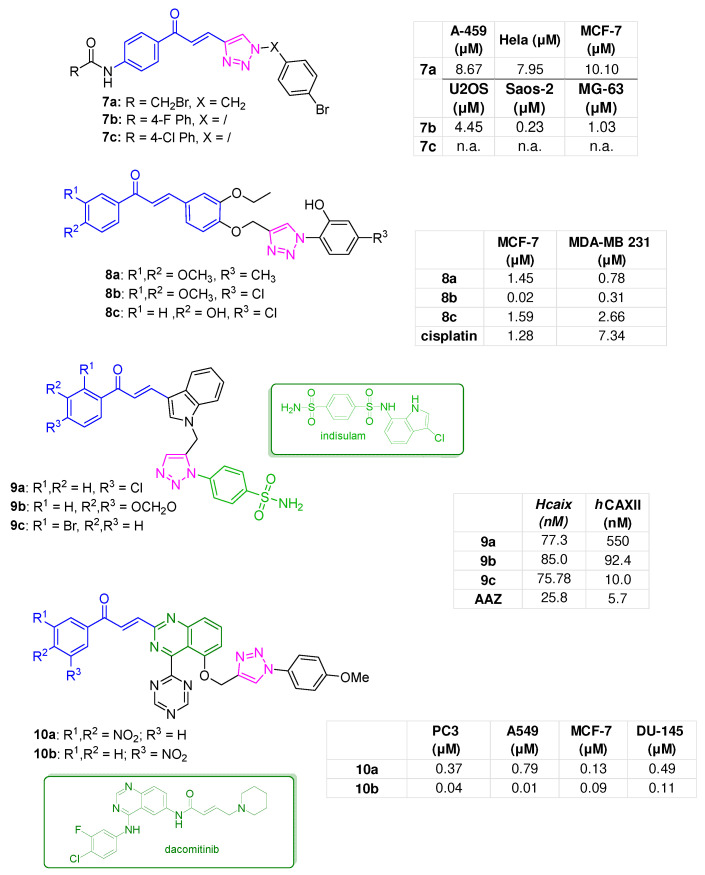
B-ring hybridisation of the chalcone scaffold (compounds **7a** [46], **7b**,**c** [47], **8a**–**c** [48], **9a**–**c** [52], **10a**,**b** [53]); bioactivities are reported in tables.

A series of chalcones characterised by a functionalised benzyl-1,2,3-triazole as the B-ring was developed by Nagarapua et al. Among them, compound **7a** (Figure 4) emerged as the most promising one, displaying a broad cytotoxic profile against four human cancer cell lines, namely MCF-7, HeLa (cervical cancer), A549, and SKNSH (human neuroblastoma), with IC_50_ values ranging from 7.41 µM to 9.76 µM. Finally, docking studies indicated that the compound can chelate the Zn^+2^ ion in the active site of histone deacetylase-8 (HDAC-8, PDB ID: 3SFH) via its carbonyl group (Table S1) [46].

In parallel, aiming to achieve tumour regression by inhibiting the phosphatidylinositol 3-kinases/mammalian target of rapamycin (PI3K/mTOR) pathways, a set of chalcones bearing a 4-bromophenyl-1,2,3-triazole moiety as the B-ring and a *para*-substituted phenyl-benzamide moiety as the A-ring were developed. Derivative **7b** (Figure 4), featuring a 4-F substituent, exhibited IC_50_ values of 4.23 and 2.30 nM against PI3K and mTOR, respectively, and substantial antiproliferative effects on human U2OS and Saos-2 osteosarcoma cells (IC_50s_ of 0.45 and 0.23 µM, respectively), MG-63 cervical cancer cells (IC_50_ of 1.03 µM), and the HeLa cell line (IC_50_ of 8.12 µM). No toxic effects (IC_50_ = 200 µM) were recorded against normal human lung cells (BEAS-2B) and a non-tumorigenic epithelial cell line (MCF-10A), highlighting a favourable selectivity. At 10 µM, this compound induced apoptosis in Saos-2 cells, confirming its anti-osteosarcoma activity. Analogue **7c** (Figure 4), with a 4-Cl atom, did not inhibit PI3K and mTOR enzymes or suppress the growth of U2OS and Saos-2 cells. [47].

Antiproliferative agents against a panel of human breast cancer cells, specifically MCF-7 and MDA-MB-231, were developed by hybridisation of a chalcone scaffold via 1,2,3-triazole with a phenol-based aryl ring. The top-performing derivatives, **8a**–**c** (Figure 4), were as potent as cisplatin (low- to sub-micromolar IC_50_ values) and exhibited a favourable selectivity index. **8b** showed IC_50_ values of 0.02 and 0.31µM againstMCF-7 and MDA-MB-23 cancel cells, respectively [48].

Among the human carbonic anhydrases (*h*CAs), the isoforms CAIX and CAXII, overexpressed in solid tumours, have been regarded as valuable anticancer targets [49]. The chalcone scaffold, previously shown to be suitable for *h*CA inhibition [50,51], was decorated with an indole heterocycle as the B-ring and linked via a 1,2,3-triazole to an arylsulfonamide moiety. The latter is regarded as a classical CA-targeting pharmacophore also found in indisulam (E7070, Figure 4), a CAIX inhibitor entered phase II clinical trials. From this design strategy, interesting analogues emerged, such as **9a** and **9b** (Figure 4), characterised by a 4-Cl- and 3,4-methylenedioxy-substituted chalcone A-ring, respectively, and both of which inhibited CAIX with nanomolar Ki values (73.3 and 85 nM, respectively). In contrast, **9c** (Figure 4), bearing a 2-Br substitution pattern, was the most potent inhibitor of the CAXII isoform (Ki = 10 nM) [52].

Given the prominent chemotherapeutic potential of dacomitinib, a quinazoline-based EGFR inhibitor (Figure 4), approved for the treatment of non-small cell lung cancer [41], Gundla et al. designed a series of chalcone-quinazoline hybrids. In particular, the triazine-quinazoline pharmacophore was decorated with a 4-methoxyphenyl fragment while different substituents were inserted in the chalcone A-ring. Derivatives **10a** and **10b** (Figure 4) showed broad antiproliferative activity against a panel of human cancer cell lines, including PC3 (prostate cancer), A549, MCF-7, and DU-145 (prostate cancer). Specifically, compound **10a**, with a 3,5-dinitrophenyl A-ring, displayed sub-micromolar IC_50_ values (IC_50s_: PC3 = 0.04 μM; A549 = 0.01 μM; MCF7 = 0.09 μM, and DU145 = 0.11 μM). On the other hand, a lowered anticancer effect was observed for the 4-nitro substituted compound **10b** (IC_50s_: PC3 = 0.37 μM; A549 = 0.79 μM; MCF7 = 0.13 μM, and DU145 = 0.49 μM). Finally, a docking study was performed for compound **10a** using tubulin crystal structure (PDB ID: 1SA0), revealing strong interactions (H-bonds, π–π stacking, and hydrophobic contacts) with key amino acids within the binding active site of colchicine, such as Ile165, Asp199, Met259, Val260, Phe262, Pro263, and Val315 (Table S1) [53].

**Figure 5:**
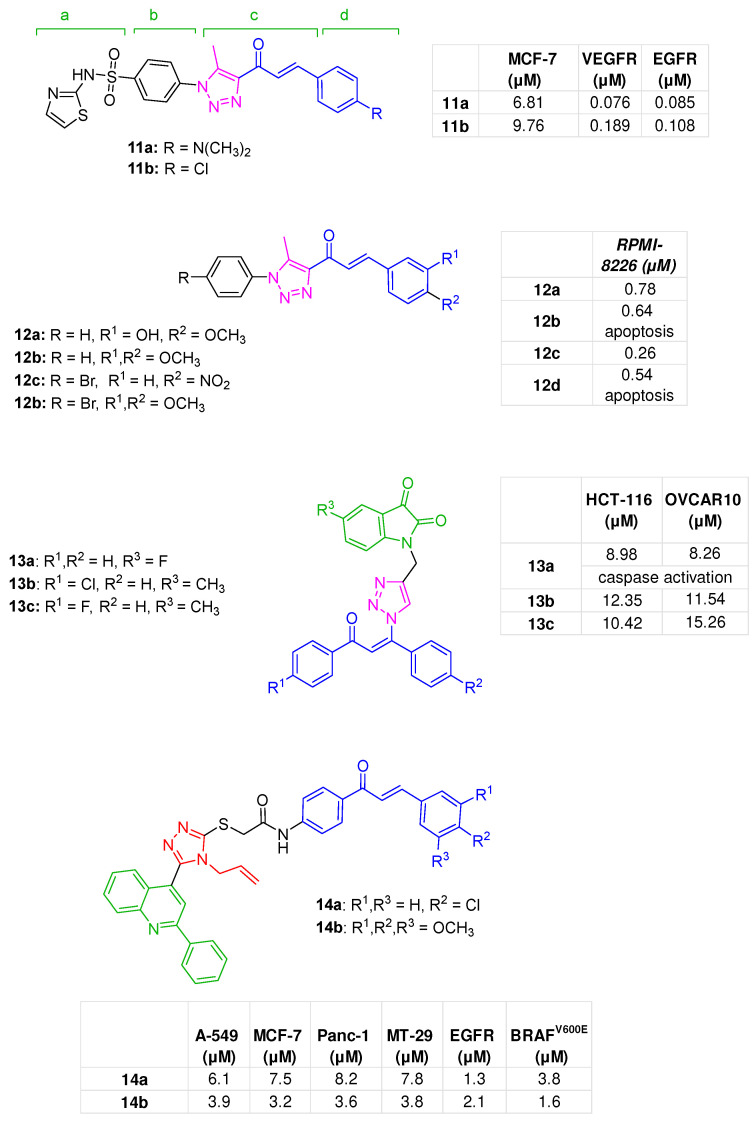
Hybrids **11a**,**b** [55], **12a**,**b** [56], **12c**,**d** [57], **13a**–**c** [58], and **14a**,**b** [59], bioactivities are reported in tables.

Given the synergistic roles of VEGFR-2 and EGFR in tumour development and angiogenesis [54], Mostafa et al. rationally developed a series of dualistic inhibitors based on a pharmacophore model for VEGFR-2 inhibition derived from the binding mode of the FDA-approved inhibitor sorafenib [44] in which four main portions (a–d) can be distinguished: (a) “ATP binding moiety”, (b) “linker”, (c) “hydrogen-bonding” moiety (interacting with the Asp-Phe-Gly (DFG) motif of the target, (d) “tail”, composed of an hydrophobic moiety. Starting from these guidelines, a new series of VEGFR-2 inhibitors was rationally designed and composed as follows: (a) a sulfathiazole synthon, (b) an aryl function, (c,d) a chalcone moiety characterised by a 1,2,3-triazole heterocycle as A-ring and a differently substituted B-ring. Compounds **11a** and **11b** (Figure 5), both bearing a *N*,*N*-dimethylamine moiety and a chlorine atom, respectively, into the *para* position of the B-ring, showed a notable antiproliferative effect against MCF-7 cells (IC_50s_ = 6.81 and 9.76 µM, respectively), thus being as potent as sorafenib (IC_50s_ = 9.18 µM). In MCF-7 cells, these analogues significantly inhibited VEGFR-2 (IC_50s_ of 0.076 and 0.189 µM for **11a** and **11b**, respectively) and EGFR (IC_50s_ of 0.085 and 0.108 µM for **11a** and **11b**, respectively), supporting an antiangiogenic effect. Molecular docking studies enabled the understanding of the binding mode of **11a** with the two kinases. Regarding VEGFR-2, the sulfonyl group interacted with Asp1046 and Lys868 through H-bonds, while a π-sulfur interaction was observed with Cys1045; the chalcone oxygen contacted Cys919; π-alkyl interactions were observed among the triazole ring and Val916, Leu1035, and Leu840 residues. Concerning EGFR binding mode, the sulfonyl oxygens engaged Asp831, Thr830, and Lys721 via H-bonds and interacted with Met742 via a π-sulfur interaction. Conversely, the triazole ring established a hydrogen bond with Met769 (Table S1). In MCF-7 cells, **11a** induced pro-apoptotic effects, resulting in both early- and late-stage apoptosis (11.53% and 19.90%, respectively). A block in the cell cycle during the S phase was also documented for this compound [55].

A small series of hybrids was designed by Selim et al., featuring a 5-methyl-1-phenyl-1*H*-1,2,3-triazole as the chalcone A-ring, while different substituents were introduced into the B-ring. In detail, analogues **12a** and **12b** (Figure 5), in which the chalcone B-ring was decorated with 3-hydroxy-4-methoxy and 3,4-dimethoxy substitution patterns, respectively, exhibited broad-spectrum cytotoxicity. Notably, both compounds strongly inhibited the proliferation of RPMI-8226 leukaemia cell line, with IC_50_ values of 0.78 and 0.64 µM, respectively, and showed good selectivity indices (SI = 45.19 and 45.53, respectively). From a mechanistic perspective, derivative **12b** induced cell cycle arrest at the G2/M phase at 6 µM concentration by triggering the mitochondrial apoptotic pathway, which involved the accumulation of reactive oxygen species (ROS), an increase in the Bax/Bcl-2 ratio, and the activation of caspases-3, -7, and -9 [56].

Another set of analogues based on the same 5-methyl-1-phenyl-1*H*-1,2,3-triazole template was reported by Abulkhair et al., among which **12c** and **12d** (Figure 5), exhibited broad antiproliferative effects, particularly against leukaemia cell lines, such as RPMI-8226 (IC_50_ values of 0.26 and 0.54 µM, respectively). In particular, in RPMI-8226 cells, **12c** demonstrated to exert the anti-leukemic effect through a pro-apoptotic mechanism, specifically by inhibiting the apoptosis-inducing factor poly(ADP-ribose) polymerase-1 (PARP-1), increasing the BAX/BCL-2 expression ratio (i.e., a decrease in the anti-apoptotic gene BCL-2, and an upregulation of the pro-apoptotic BAX gene), and upregulating caspases-3 and -9. A cell cycle arrest in the S-phase and a favourable safety profile (SI = 12.19) were also observed [57].

The 1*H*-indole-2,3-dione (isatin) pharmacophore, a “privilege synthon” widely exploited in drug discovery [52], was introduced into the β-position of a chalcone unsaturated carbonyl moiety, through a 1,2,3-triazole linker, to obtain a series of hybrids endowed with antiproliferative activity. The structure-activity relationship (SAR) study performed identified **13a**–**c** (Figure 5) as the most potent cytotoxic agents in the series, particularly against colon HCT-116 and ovarian OVCAR-10 cancer cell lines, more active than the reference compounds cisplatin and 5-FU. In detail, **13a**, with an unsubstituted chalcone scaffold and a 5-fluoro isatin, exhibited low-µM IC_50_ values against HCT116 and OVCAR10 cells (IC_50s_ = 8.98 and 8.26 μM, respectively) and a favourable SI (2.13 for HCT-116 and 2.32 for OVCAR-10) superior to the reference drugs (5-FU: SI = 0.95 and cisplatin: SI = 1.37). Apoptosis studies conducted at 10 μM (HCT-116 cells) revealed a significant increase in the number of apoptotic cells after 48 h of treatment (42.65%), with activation of caspases-3 and -7. Finally, docking analysis was used to examine how **13a** binds to the selected potential targets VEGFR-2 and EGFR. In the VEGFR-2 active site, **13a** formed a hydrogen bond between the isatin carbonyl and Arg1027, with the chalcone ring B fitting into a hydrophobic pocket of the enzyme. For EGFR, π-π interactions were observed between the aromatic rings of the chalcone scaffold and the Tyr318 and His193 residues in the enzyme’s active site (Table S1) [58].

A series of quinoline-chalcone hybrids bearing a 1,2,4-triazole linker was designed to develop antiproliferative agents targeting EGFR and BRAF^V600E^ kinases. Among the series, compounds **14a** and **14b** (Figure 5), both featuring an allyl-substituted triazole, emerged as the most effective agents, exhibiting broad and potent antiproliferative activity across multiple cancer cell lines, i.e., A-549, MCF-7, Panc-1, and HT-29, with low-micromolar IC_50_ values. A potent inhibition of BRAF^V600E^ (IC_50s_ of 3.8 µM for **14a** and 1.6 µM for **14b**) and EGFR-TK (IC_50s_ of 1.3 µM for **14a** and 2.1 µM for **14b**) also emerged, supported by molecular docking studies that demonstrated an appropriate fit of the hybrids into the active sites of the studied kinases (Table S1). Analogue **14a** also caused cell cycle arrest at G2/M transition and a significant induction of apoptosis [59].

### 3.2. Chalcone Pyrazole Hybrids

**Figure 6:**
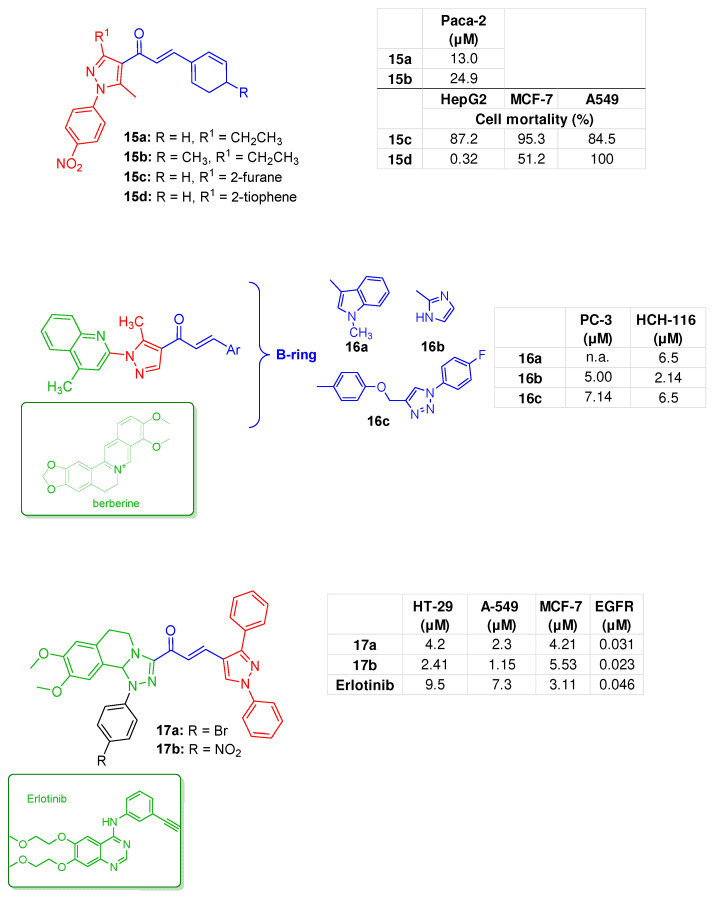
Chalcone-pyrazole hybrids **15a**,**b** [60], **15c**,**d** [61], **16a**–**c** [62], **17a**,**b** [64]; bioactivities are reported in tables.

A library of chalcones characterised by a 3-ethyl-5-methyl-1-(4-nitrophenyl)-1*H*-pyrazole moiety as the A-ring was developed by Hassaneen et.al. for pancreatic cancer treatment. In detail, derivatives **15a** and **15b** (Figure 6), characterised by a benzene or a tolyl function as B-ring, emerged as the most representative due to their ability to inhibit pancreatic cancer (PaCa-2) cells proliferation in vitro, showing IC_50_ values of 13.0 and 24.9 µM, respectively, comparable to that of doxorubicin (28.3 µM). The mechanism underlying the anticancer effects of these analogues involved the activation of caspases-3 and -8, the upregulation of the anticancer-related gene cyclin-dependent kinase 6 (CDK6), and the induction of the DNA damage response. Analogue **15a** also demonstrated the ability to induce cell cycle arrest at the S and G2/M phases [60].

Moreover, another series of hybrids was developed in which the pyrazole, as the chalcone A-ring, was functionalised at the 3-position with a 5-membered heterocycle. Analogues **15c** and **15d** (Figure 6), which feature a furan and a thiophene moiety, respectively, and an unsubstituted benzene as B-ring, demonstrated a broad antiproliferative effect. Compound **15d** was the most promising, with IC_50_ values of 27.7 µg/mL (A549 cells) and 26.6 µg/mL (HepG2 cells), proving to be as potent as doxorubicin. This analogue was found to reduce the expression levels of genes associated with lung and liver cancers. Furthermore, in HepG2 cells, treatment with **15d** increased DNA damage and DNA fragmentation. [61].

A series of chalcones was designed with the (5-methyl-1*H*-pyrazol-1-yl)quinoline pharmacophore as the chalcone A-ring, resulting in **16a**–**c** (Figure 6), which showed good antiproliferative activity against HCT-116 colon cancer cells (IC_50_ values ranging from 2.1 µM to 6.50 µM). Moreover, **16b** and **16c** also exhibited considerable effects against prostate PC-3 cancer cells (IC_50s_: 5.00 and 7.14 µM, respectively). Unfortunately, these derivatives did not possess a favourable safety profile, as evidenced by the remarkable antiproliferative effect observed in normal breast epithelial cell lines (FR 2). The binding mode of **16b** to EGFR was studied, and it was shown that the pyrazole and imidazole rings interacted with Asp831 via anion-π interactions, and that the imidazole ring also interacted with Phe699. The quinoline moiety interacted with Leu694, Leu820 and Val702, with the methyl group contacting Ala719 (Table S1) [62].

The tetrahydroisoquinoline (THiQ) heterocycle, found in several NPs with EGFR-inhibitory properties, such as berberine ([63], inspired the rational design of a series of chalcone-based hybrids characterised by a fused *N*1-aryl-1,2,4-triazole-THiQ moiety as the A-ring and a 1,3-diphenyl-1*H*-pyrazole B-ring. Derivatives **17a** and **17b** (Figure 6) showed notable antiproliferative effects with low micromolar IC_50_ values against HT-29 (colorectal adenocarcinoma), A549, and MCF-7 cell lines, demonstrating potency comparable to that of erlotinib. These hybrids also effectively inhibited total EGFR (IC_50_ values of 0.031 µM and 0.023 µM for **17a** and **17b**, respectively). Moreover, **17b** was the most active against the mutated EGFR proteins EGFRL858R and EGFRT790M, with IC_50_ values of 34.78 µM and 199.53 µM, respectively. EGFR inhibition was confirmed by molecular docking studies, which demonstrated a high affinity of **17b** for the EGFR binding pocket through key interactions, including an H-bond with Met742, a π-H interaction with Lys721, and pi-cation interactions with Lys721 (Table S1). In A549 cells, both hybrids, tested at their respective IC_50s_, induced apoptosis and necrosis, and arrested the cell cycle in G2/M and pre-G1 phases [64].

## 4. Curcuminoids

**Figure 7:**
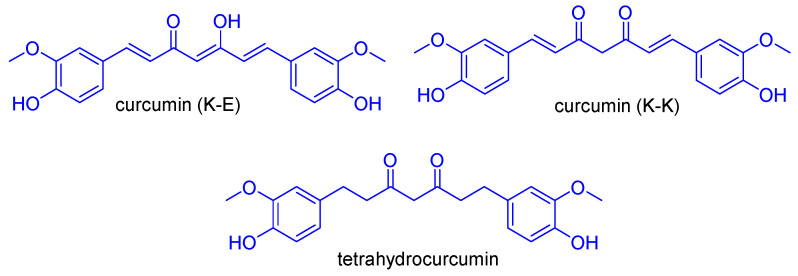
Structures of curcumin’s tautomer and tetrahydrocurcumin [68,69].

The polyphenol curcumin ((1*E*,6*E*)-1,7-bis(4-hydroxy-3-methoxyphenyl)hepta-1,6-diene-3,5-dione, the main bioactive component of the rhizomes of *Curcuma longa* L., is among the most extensively studied NPs, with established efficacy and safety profiles for the prevention and treatment of various multifactorial disorders, including cancer and neurodegeneration. In particular, curcumin demonstrated a wide range of anticancer effects both in vitro and in vivo, inhibiting cancer cell proliferation, metastasis, and invasion, and inducing apoptosis [65]. The molecular mechanisms underlying curcumin’s antitumor potential involve the ability to interfere with various targets and signalling pathways, such as NF-κB, TNF (Tumour Necrosis Factor)-α, and signal transducer and activator of transcription 3 (STAT3) [66]. The safety profile of curcumin has been primarily demonstrated through clinical trials [67]. The curcuminoid pharmacophore consists of a mixture of tautomers that can interconvert, specifically the asymmetric β-keto-enol and the symmetric diketo structures (K-E and K-K, respectively, Figure 7). The K-E tautomer is the most prevalent form because the intramolecular H-bond stabilises the central structure fragment. The versatility of this scaffold is underscored by the extensive literature on curcumin-inspired analogues as anticancer agents [68,69].

### 4.1. Curcumin-1,2,3-triazole Hybrids

**Figure 8:**
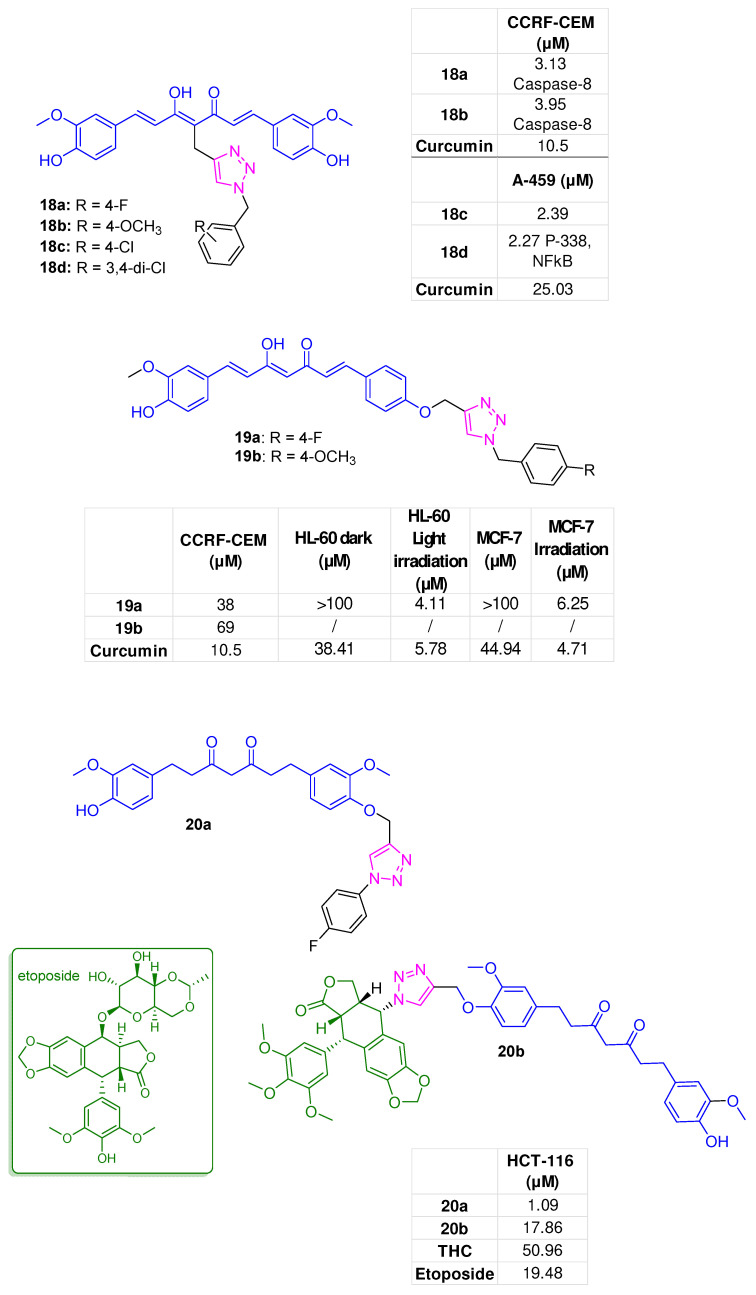
1,2,3-triazole hybrids corresponding to curcumin and THC (**18a**,**b** [70], **18c**,**d** [71], **19a**,**b** [70,72], **20a** [73], **20b** [74]); bioactivities are reported in tables.

Seghetti et al. functionalized the curcumin template at different positions with a *para*-fluorine or *para*-methoxy benzyl moiety via a 1,2,3-triazole spacer to investigate the structural requirements of this scaffold for developing effective anticancer agents. Specifically, by retaining the entire curcumin structure, decoration of the central C-4 position of the heptadienone fragment produced compounds **18a** and **18b** (Figure 8) as a mixture of K-K and K-E tautomers. These derivatives exhibited notable antiproliferative effects on the T-acute lymphoblastic leukaemia cell line (CCRF–CEM) with IC_50_ values of 3.13 and 3.95 µM, respectively, and were more effective than curcumin itself (IC_50_ = 10.5 µM). The induction of apoptosis was the main molecular mechanism by which analogue **18a**, at 3 µM, caused apoptosis in CCRF–CEM cells as evidenced by decreased mitochondrial transmembrane potential and marked activation of caspase-8, thus affecting both the intrinsic (mitochondria-related) and extrinsic cell-death pathways [70].

Functionalization of the C-4 central position of the curcumin scaffold also afforded derivatives **18c** and **18d** (Figure 8), with 4-Cl and 3,4-di-Cl substitution patterns on the benzyl moiety, respectively, that inhibited the proliferation of A549 lung cancer cells at low micromolar potencies (IC_50_ = 2.93 and 2.27 μM, respectively). Once more, the functionalization of the C-4 position of curcumin yielded hybrids more effective than curcumin itself (IC_50_ = 25.03 μM). Regarding the mechanism underlying the anticancer effect of **18d**, an increase in phosphorylation of p38, c-Jun N-terminal kinase (JNK), and extracellular signal-regulated kinase (ERK), along with a decrease in NF-kB expression and an upregulation of nuclear factor IkBα, was reported. Moreover, in a zebrafish xenograft tumor model, this analogue, at a 2.5 μM concentration, showed a consistent decrease in tumor focal areas, while little effect was recorded on normally developing cells (zebrafish embryos) [71].

On the contrary, when the same modification involved one aryl side group, derivatives **19a** and **19b**, obtained as K-E tautomers (Figure 8), exhibited markedly reduced cytotoxicity (IC_50_ = 38 and 69 µM, respectively) compared to the C4-functionalized counterparts **18a** and **18b**. The above data indicated that the simultaneous presence of both 4-hydroxy-3-methoxyphenyl side groups is crucial for achieving optimal anticancer effects. Interestingly, in a follow-up study, **19a** was identified as a promising photosensitizer agent, suitable for photodynamic anticancer therapy. Indeed, photoactivation of **19a** by exposure to low-irradiance white LED light led to a significant enhancement of cytotoxicity against promyelocytic leukaemia HL-60 and MCF-7 cancer cells (IC_50_ = 4.11 and 6.25 µM, respectively); these effects were mediated by pro-apoptotic and pro-ferroptotic mechanisms, as demonstrated by increased PARP cleavage and decreased GPX4 expression [72].

### 4.2. Tetrahydrocurcumin-Hybrids

Decoration of one aryl side of tetrahydrocurcumin (THC) with a para-fluorophenyl group via a 1,2,3-triazole yielded **20a** (Figure 8). This compound showed consistent anticancer activity against human colon carcinoma HCT-116 cells, with an IC_50_ of 1.09 µM, and caused cell cycle arrest at the G1 phase, before DNA synthesis. Conversely, only weak effects were observed on A549 and HepG2 cells. The binding of **20a** with the APC-Asef protein, known for its key role in the development of colon cancer, was studied using molecular docking and several H-bonding and hydrophobic interactions were observed. In particular, Asp459, Glu460, Arg463, and Arg549 were involved. Asp459 and Phe510 interacted with the benzene and triazole moieties via hydrophobic interactions (Table S1) [73].

The THC scaffold was also connected via a 1,2,3-triazole to a synthon related to podophyllotoxin (PPT), an NP known for its essential role in cancer therapy. [63]. The THC-PPT hybrid **20b** (Figure 8) demonstrated an antiproliferative activity against HCT-116 cells with an IC_50_ of 17.86 µM. It was more effective than THC (IC_50_ = 50.96 µM) and showed comparable efficacy to etoposide (IC_50_ = 19.48 µM) [74].

## 5. Monocarbonyl Curcumin Analogues

Different strategies have been employed to address some curcumin-related issues, specifically its limited chemical stability and poor aqueous solubility, which impair bioavailability and restrict its therapeutic window [64]. For instance, simplification of the central curcumin 5-hydroxy-1,7-diarylhepta-1,4,6-trien-3-one core by replacing it with a 1,4-pentadiene-3-one fragment resulted in the C5-curcumin analogue family (BAPD, Figure 1A). Michael’s reactivity has been reported as a potential feature underlying the bioactivity of this class (Figure S1) [75].

### 5.1. C-5-Monocarbonyl Curcumin-1,2,3-triazole Hybrids

**Figure 9:**
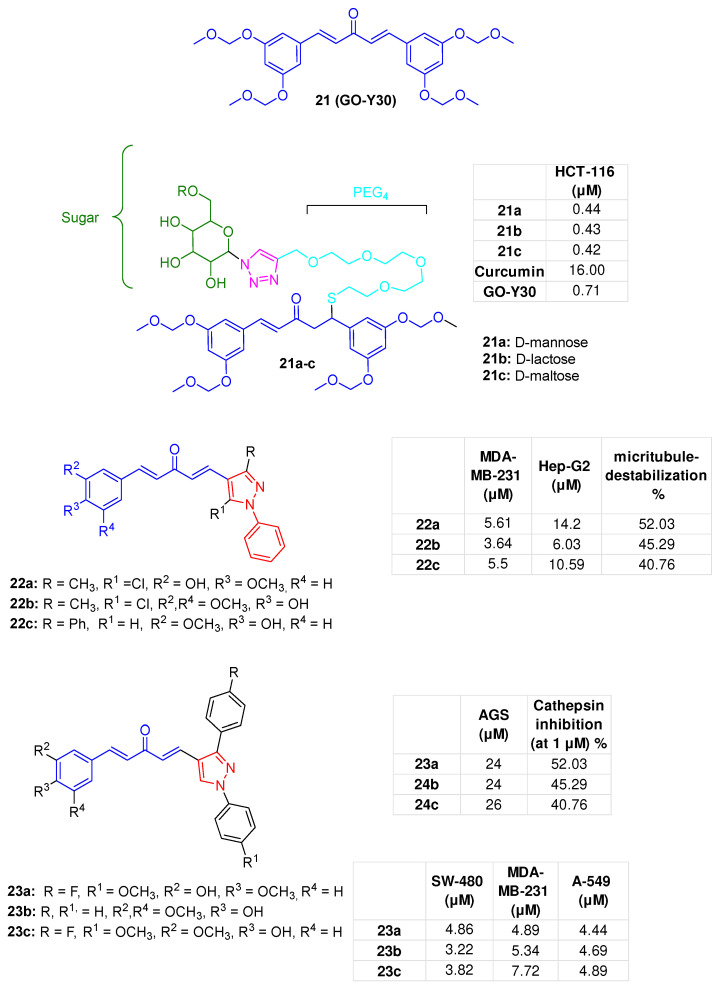
Triazole and pyrazole hybrids based on the BAPD scaffold (**21** [76], **21a**–**c** [78], **22a**–**c** [79], **23a**–**c** [80]); bioactivities are reported in tables.

The monocarbonyl-C5-curcuminoid GO-Y030 (**21**, Figure 9), recognised for its anticancer properties [76,77], was converted into a Michael adduct by attaching a hydrophilic thiol-based linker composed of a polyethene glycol (PEG) unit connected via a 1,2,3-triazole with a sugar moiety. The designed glycoconjugates (**21a**–**c**, Figure 9), which contain D-mannose, D-lactose, and D-maltose sugar units, respectively, can be considered prodrugs because they can undergo a retro-thia-Michael reaction with intracellular thiols, particularly glutathione, leading to the formation of the parent compound **21**. In HCT-116 cells, **21a**–**c** showed low-micromolar antiproliferative potencies (IC_50_ = 0.42–0.55 µM range), superior to that of curcumin (IC_50_ = 16 µM) and comparable to that of the lead **21** (IC_50_ = 0.72 µM) [78].

### 5.2. C-5-Monocarbonyl-curcumin-pyrazole-hybrids

Truong and colleagues created a series of asymmetric BAPD analogues **22a**–**c** (Figure 9), with one side of the pentadienone tether bearing an *N*1-aryl-1*H*-pyrazole group and the other side featuring a phenyl ring with electron-rich substituents such as alkoxy and hydroxy groups. The compounds effectively inhibited the growth of MDA-MB-231 (IC_50_ range: 2.43−7.84 μM) and HepG2 (IC_50_ range: 4.98−14.65 μM) cancer cells. Moreover, in MDA-MB-231 cells, they triggered apoptosis at 1 μM, induced G2/M phase cell cycle arrest at 5 μM, and elevated caspase-3 activity at 10 μM. At 20 μM, microtubule-destabilizing effects ranging from 40.76% to 52.03% were observed [79].

The same authors also developed another set of BAPD hybrids (**23a**–**c**, Figure 9) bearing a 1,3-diphenyl-1H-pyrazole moiety. Derivatives demonstrated significant cytotoxicity (low micromolar IC_50_ values) against three human cancer cell lines that overexpress EGFR, namely colon cancer SW480, MDA-MB-231, and A549. Treating SW480 cells with 10 μM of **23b** caused 13.26% of the cells to undergo apoptosis, with a corresponding increase in caspase-3 activity 2.37-fold. Additionally, the percentage of cells in the G2/M phase consistently rose to 21.90% and 22.16%. Finally, **23b**, at 5 µM, inhibited EGFR-related colorectal cancer forms, such as EGFR^WT^ and EGFR^L858R^ (30% and 35% inhibition, respectively) [80].

## 6. Aurones

Aurones (2-arylidenebenzofuran-3-(2*H*)-one, Figure 1A) are a class of NPs related to flavonoids that have been shown to exhibit diverse biological activities, including anticancer, anti-Alzheimer’s, and anti-inflammatory effects, making them valuable privileged structures in medicinal chemistry. The electrophilic α,β-unsaturated carbonyl fragment (Figure S1) not only imparts unique physicochemical characteristics but also crucially influences their biological activities by modulating various enzymes and receptors. A relevant anticancer potential has also been demonstrated, prompting medicinal chemists to exploit this scaffold to develop effective anticancer agents [81,82].

### 6.1. Aurone-1,2,3-triazole Hybrids

**Figure 10:**
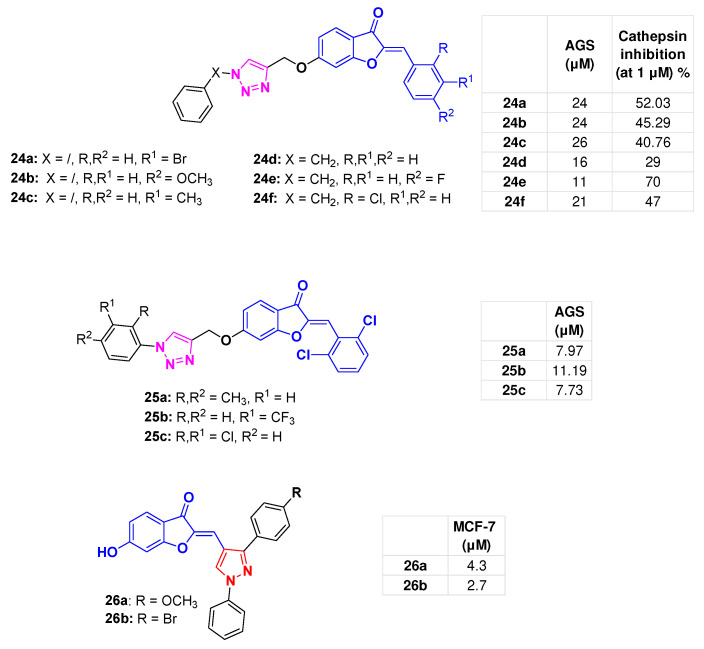
Aurone-1,2,3-triazole and pyrazole hybrids **24a**–**c** [83], **24d**–**f** [85], **25a**–**c** [86], **26a**,**b** [87]; bioactivities are reported in tables.

The study by Kumar et al. involved functionalising the aurone core by inserting a 1-phenyl-1,2,3-triazole group, via an oxymethylene spacer, at the 6-position of the benzofuran-3-one nucleus, while various substituents were introduced into the arylidene aromatic ring. Compounds **24a**–**c** (Figure 10) were identified, which showed a two-digit micromolar inhibitory potency against the proliferation of the gastric adenocarcinoma (AGS) cell line (IC_50_ = 24–26 µM range). Furthermore, **24a** and **24b** at 1 µM inhibited (by 42% and 36%, respectively) cathepsin B [83], a cysteine protease that plays a key role in cancer progression and apoptosis [84].

The above study was further expanded with the design of a set of analogues, in which a benzyl was introduced into the 1*H*-1,2,3-triazole instead of the corresponding phenyl, leading to the identification of **24d**, **24e**, and **24f** (Figure 10), which inhibited AGS cells’ proliferation (IC_50_ = 16 μM, 11μM, and 21 μM, respectively). The data suggested a favourable effect of a halogen atom in the *para*-position of the arylidene aurone moiety due to an inductive effect (see **24e** and **24f**). Analogue **24e**, tested at 10 µM, inhibited cathepsin B by 70%, while lower inhibition percentages were observed for **24d** and **24f** (29% and 47%, respectively). Molecular docking studies, conducted to assess the ability of the designed hybrids to interact with the active site of cathepsin B, revealed that the compounds bound to the protein’s active site. In particular, the observed nucleophilic attack of the Cys29 thiol group on the aurone α,β-unsaturated carbonyl moiety resulted in the formation of a stable tetrahedral intermediate, thus suggesting an active site-directed enzyme inhibition [85].

Kumara et al. designed a further series of aurone-based derivatives (**25a**–**c**, Figure 10), bearing a 2′-6′-di-Cl decorated benzylidene moiety and different substituents on the 1*H*-1,2,3-triazole phenyl function, inserted at the 6-position of the benzofuran-3-one scaffold (Figure 10). Good antiproliferative activity was observed in AGS cells, with compound **25a** showing an IC_50_ of 7.97 μM, **25b** of 11.19 μM, and **25c** of 7.73 μM. Compound **25c** was computationally analysed against the HER2 receptor, yielding a docking score of −4.88. It established H-bond interactions with Cys805 and Thr862, as well as a π-cation interaction with Lys753 (Figure S1) [86].

### 6.2. Aurone-Pyrazole Hybrids

Aurones **26a**,**b** (Figure 10) featuring a diaryl-pirazole and a hydroxyl group at the 6-position of the benzofuran-3-one core, showed a remarkable antiproliferative effect on MCF-7 cells (IC_50s_ of 4.3 and 2.7 µM, respectively) [87].

## 7. 3,5-Bis(arylidene)-4-piperidones

**Figure 11:**
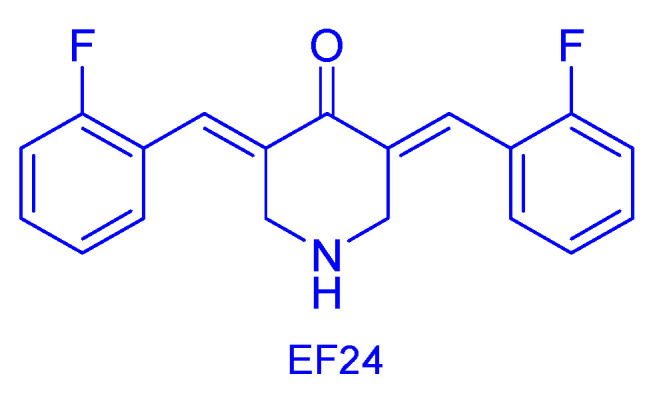
Structure of EF24 [89] as an anticancer agent.

Among the curcumin mimics, an interesting class of bioactive compounds is represented by the 3,5-bis(arylidene)-4-piperidone (BAP, Figure 1A), which has been widely exploited for the design of anticancer agents [88]. In particular, 3,5-bis(2-fluomethoxybenzylidene)-4-piperidone (EF24, Figure 11) has emerged as a valuable anticancer agent with broad antiproliferative effects (MDA-MB-231 and PC3 cell lines) and the ability to inhibit hypoxia-inducible factor, an intracellular pro-angiogenic transcription factor [89]. The versatility of the BAP pharmacophore allowed for various modifications at the side aryl rings and the piperidone nitrogen atom; the latter is particularly suitable for hybridisation. This class of compounds is classified as Michael acceptors able to interact, under physiological conditions, with nucleophiles of biological macromolecules (Figure S1).

### 3,5-Bis(arylidene)-4-piperidone-1,2,3-triazole Hybrids

**Figure 12:**
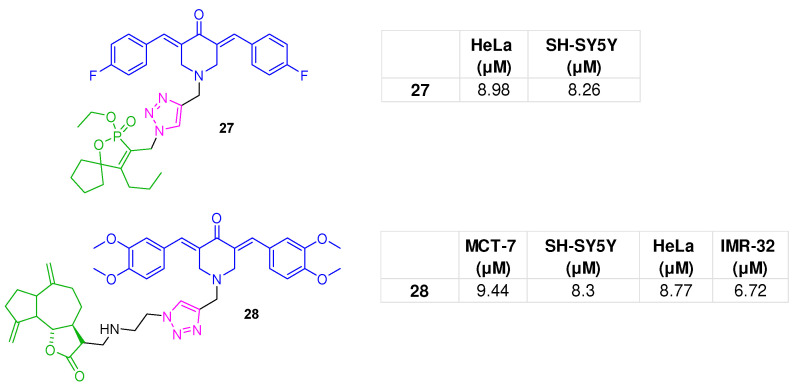
BAP-1,2,3-triazole hybrids **27** [90] and **28** [91]; bioactivities are reported in tables.

The BAP scaffold, bearing 4-F-phenyl side rings, was linked via a triazole to a 1,2-oxaphospholene-based pharmacophore at the N atom, affording compound **27** (Figure 12), which demonstrated potent antiproliferative effects against A549 and neuroblastoma (SH-SY5Y) cell lines, while modest effects were observed against Hep-G2 and HeLa cells. This analogue at 30 µM suppressed basal anaerobic glycolysis in HeLa cells, likely by inhibiting lactate production. Furthermore, under the same experimental conditions, it reduced the glycolytic reserve, indicating an influence on abnormal metabolism, such as glycolysis, a distinctive feature of cancer cells, also known as the Warburg effect. [90].

From the study by Brel et al., hybrid **28** (Figure 12), designed by combining a bis-3,5-demethoxyarylidene-4-piperidone framework with a sesquiterpene lactone pharmacophore via a 1,2,3-triazole linker, was identified as an antiproliferative agent active against a broad panel of cancer cell lines (MCF-7, SH-SY5Y, HeLa, and IMR-32). It also disrupted glycolytic metabolism of tumor cells, thus reducing the extracellular acidification rate related to glycolysis (by 34.30%), glycolytic capacity (by 56.40%), and glycolytic reserve (by 91.79%). In silico screening identified compound **28** as a potential inhibitor of pyruvate kinase M2 (PKM2), a key allosteric enzyme in glycolysis and a significant cancer-related target. In particular, the key interactions at the phosphoenolpyruvate binding site of PKM involved Ser362, Thr328, and Ile51 (Table S1) [91].

## 8. Conclusions

The development of an effective anticancer agent often depends on a hybridisation strategy that integrates selected biologically relevant motifs into a single molecular entity to optimise therapeutic effects. This approach has shown potential to optimise anticancer activity by enhancing antiproliferative effects against cancer cell lines while generally maintaining low toxicity towards normal cells. For instance, it supports the development of compounds with well-defined mechanisms of action, including modulation of specific molecular targets or multiple molecular pathways involved in the onset and progression of cancer. In this context, five-membered nitrogen heterocycles, such as triazoles and pyrazoles, have played a key role in enabling the design of hybrid molecules, likely due to their ability to strengthen interactions with the molecular targets. Likewise, NPs have also provided a valuable source of lead compounds and biologically relevant templates, with scaffolds bearing electrophilic α,β-unsaturated carbonyl systems playing a significant role. Indeed, chalcones, curcuminoids, aurones, and related derivatives, namely 1,5-bis(aryl)-1,4-pentadiene-3-ones (BAPDs) and 3,5-bis(arylidene)-4-piperidones (BAPs), have emerged as crucial platforms for the development of novel therapeutic agents through tailored structural modifications. By integrating these electrophilic frameworks with a triazole or pyrazole heterocycle, valuable anticancer agents have been developed, sometimes characterised by multitarget effects (Table S2).

Interesting results were achieved by incorporating an anticancer pharmacophore (**2b**, **4a**–**c**, **6c**, **9c**, **20b**) or a “privileged synthon” (**11a**,**b**, **13a**, **21**, and **28**).

Compound **2b** (a chalcone-altretamine hybrid) exhibited potent inhibitory activity against HCT-116 cell proliferation (IC_50_ = 0.036 µM), also demonstrating the capability to selectively inhibit the MMP-10 enzyme (IC_50_ = 0.18 µM). Furthermore, derivatives **4a**–**c** (chalcone-ciprofloxacin hybrids: IC_50s_ range of 2.53–4.32 µM on HCT-116 cells) demonstrated a multitarget effect by inhibiting topoisomerases and tubulin polymerisation, triggering DNA damage in HCT-116 cells and causing G2/M cell cycle arrest. Derivative **6c** (chalcone-protoapigenone hybrid: IC_50_s ranging from 0.20 to 0.32 µM and 0.25 to 0.51 µM on MDA-MB-231 and MCF-7 cells, respectively) proved to negatively affect DNA synthesis and to induce apoptosis by increasing caspase-3 activity. The conjugation of the indeno-chalcone scaffold with an aryl sulphonamide moiety, as CA-targeting pharmacophores, enabled the development of **9c** as a potent CAXII inhibitor (Ki = 10 nM). The THC-PPT hybrid **20b** showed an antiproliferative activity superior to the corresponding synthon. The chalcone-sulfathiazole hybrids **11a**,**b** demonstrated to affect the growth of MCF-7 cells. (IC_50s_ = 6.81 and 9.76 µM, respectively), to inhibit VEGFR (IC_50_s = 0.076 and 0.189 µM, respectively) and EGFR (IC_50s_ = 0.085 and 0.108 µM, respectively). These outcomes further support the VEGFR-2 pharmacophore model for the design of kinase inhibitors. Compound **13a** (chalcone-isatin hybrid) showed a broad-spectrum cytotoxicity, achieved through induction of apoptosis and modulation of the cell cycle. Conjugation of a BAP-based synthon with an NP-derived sesquiterpene afforded **28**, endowed with a wide cytotoxicity profile (low-micromolar IC_50_ values against MCF-7 and HeLa cell lines), likely due to glycolysis-inhibiting effects. An interesting design strategy encompassed the development of the prodrugs **21a**–**c**, which behave as PEG-glycoconjugated thia-Michael adduct of the C-5-monocarbonyl curcuminoid GO-Y030 (**21**), and showed a significant antiproliferative effect against HCT-116 cells (IC_50_ = 0.42–0.44 µM). Notably, their capacity to react with intracellular thiols suggests they act as prodrugs, underscoring their potential as bioresponsive anticancer agents.

Moreover, the quinoline-based privileged template, widely present in EGFR inhibitors, was extensively and successfully exploited to design hybrids (i.e., **14a**,**b**, **16b**, **17a**,**b**). Hybrids **14a**,**b**, bearing a 1,2,4-triazole spacer, exhibited broad-spectrum antiproliferative effects (low-micromolar IC_50s_ against a panel of cancer cells, including MCF-7, A-549, and MT-29). At the molecular level, induction of apoptosis, modulation of the cell cycle (G2/M arrest), and inhibition of the kinases EGFR and BRAF^V600E^ (IC_50s_ = 1.3 and 2.1 µM, respectively) were observed. Hybrid **16b** affected HCT-116 cell growth (IC_50_ = 2.14 µM); while the hybrids **17a**,**b** with a THiQ synthon, proved to effectively inhibit the growth of different cancer cell lines, namely HT-29, A-549, and MCF-7 (IC_50s_ values ranging from 1.15 to 5.5 µM) as a consequence of an inhibitory effect on EGFR (IC_50s_ = 0.031 and 0.23 µM, respectively). Hybrids **10a**,**b** have also been developed as broad-spectrum antiproliferative agents (sub-micromolar IC_50s_ against PC3, A549, MCF-7, and DU-145 cell lines).

The *para*-fluorine aryl substitution pattern enabled the development of promising anticancer agents (**18a**, **19a** and **20a**). Indeed, by applying the click-chemistry strategy to the central C-4 position of the curcumin scaffold, **18a** was designed to inhibit the proliferation of CCRF-CEM cells (IC_50_ = 3.13 µM) and to trigger programmed cell death by inhibiting mitochondrial transmembrane potential and activating caspase-8. On the contrary, when the same functionalisation was performed on one aryl moiety of curcumin, hybrid **19a**, a potent photoinducible cytotoxic agent, was obtained. Derivative **20a** (THC/4-F benzene hybrid) showed selective cytotoxicity against the HCT-116 cell line (IC_50_ = 1.09 µM), likely due to the capability of inducing cell cycle arrest in G1.

Furthermore, poly-methoxylated aryl groups were extensively utilised due to their behaviour as privileged structures. For instance, 3,4,5-trimethoxy and 3,4-dimethoxy aryl rings, which were incorporated into hybrids **4c**, **5a**, **6c**, **14b**, **8a**,**b**, and **12b**,**d**, enabled the development of broad antiproliferative agents (ICT-116, SSC-25, MDA-MB-231, and MCF-7 cancer cell lines). On the contrary, BAPD functionalised with a 3,5-dimethoxy-4-hydroxyphenyl moiety yielded hybrid **23b**, which inhibited the proliferation of MDA-MB-231 and MCF-7 cancer cells (IC50s = 5.34 and 4.69 µM, respectively). At the molecular level, induction of apoptosis (increase in caspase-3 activity), G2/M cell-cycle arrest, microtubule destabilisation, and inhibition of EGFR (30% at 5 µM) were observed.

Together, these studies highlighted the importance of integrating a triazole or pyrazole heterocycle with an electron-rich NPs-inspired template as a strategic approach for obtaining hybrids capable of influencing cancer-related mechanisms.

## 9. Future Perspectives

The molecular hybridisation design strategy, using the “fragment-linking” approach, i.e., combining suitable anticancer pharmacophores, has successfully enabled the development of anticancer agents.

To this aim, the widespread exploration of privileged structures, often involving NPs, has been pursued. The significant results support the idea that a compound’s bioactivity can vary with context and can be adjusted to meet specific therapeutic objectives, thereby challenging the usual assumptions behind PAINS. Five-membered nitrogen-containing heterocycles, such as triazoles and pyrazoles, played a pivotal role in the development of bioactive molecules. They are appreciated not just for their ability to participate in different non-covalent interactions with molecular targets, but also for the structural flexibility they provide to the entire molecule. On the other hand, electrophilic NPs have been essential scaffolds in the rational design of anticancer agents. Moving forward, the strategic hybridisation of these fragment classes could further prove its crucial role in developing the next generation of anticancer drug candidates with improved bioactivity profiles.

## Data Availability

Data are contained within the article and Supplementary Materials.

## Supplementary Materials

The following supporting information can be downloaded at: https://www.mdpi.com/article/10.3390/molecules31020355/s1, Figure S1: Michael Addition reaction of a thiol-based moiety to the electron-rich fragment of chalcone or curcumin main templates; Table S1: Docking Studies of the lead compounds described in the present review; Table S2: The best-performing hybrid molecules are described in the present review.

## Abbreviations

The following abbreviations are used in this manuscript:

ATR	ataxia-telangiectasia and Rad3-related protein
BAP	3,5-bis(arylidene)-4-piperidone
BAPD	1,5-bis(aryl)-1,4-pentadiene-3-one
Ca	carbonic anhydrase
Chk1	checkpoint kinase 1
CP	ciprofloxacin
CRC	colorectal cancer
CuAAC	Cu-catalysed azide-alkyne cycloaddition
EGFR	epidermal growth factor receptor
ERK	extracellular signal-regulated kinase
HDACs	histone deacetylases
HNSCCs	head and neck squamous cell carcinomas
JNK	c-Jun N-terminal kinase
K-E	β-keto-enol
K-K	diketo
MMPs	Matrix metalloproteinases
NF-κB	nuclear factor-kB
NPs	Natural products
PAINS	pan-assay interference compounds
PARP-1	poly(ADP-ribose) polymerase-1
PEG	polyethene glycol
PKM2	pyruvate kinase M2
PPT	podophyllotoxin
ROS	reactive oxygen species
STAT3	signal transducer and activator of transcription 3
THC	tetrahydrocurcumin
THiQ	tetrahydroisoquinoline
VEGFR	vascular endothelial growth factor receptor
**Cancer cells lines**	
A549	lung cancer
AGS	gastric adenocarcinoma
Capan-1	pancreatic cancer
CCRF–CEM	T-acute lymphoblastic leukaemia cell line
Detroit 562	squamous carcinoma
DU-145	prostate cancer
HCT-116	colon carcinoma
HEK293	human embryonic kidney
HeLa	cervical cancer
HepG2	human hepatoma carcinoma
HL-60	promyelocytic leukaemia
HT-29	colorectal adenocarcinoma
MCF-7	human breast cancer
MDA-MB-231	human breast cancer
MG-63	cervical cancer
OVCAR-10	ovarian cancer
PC3	prostate cancer
RPMI-8226	leukaemia
Saos-2	osteocarcinoma
SH-SY5Y	neuroblastoma
SKNSH	human neuroblastoma
SSC-25	tumorigenic keratinocyte
SW480	colon cancer
U2OS	osteocarcinoma
