# Supplementary material for: Triazole and Pyrazole Hybrids of Electrophilic Natural Products as Promising Anticancer Agents
Source: Molecules. 2026 Jan 19;31(2):355. doi: 10.3390/molecules31020355 (PMC12843775; doi:10.3390/molecules31020355)
Supplement: Supplementary file 1 [file molecules-31-00355-s001.zip › molecules-4048187-supplementary.pdf]

molecules

Supplementary Materials

Triazole and Pyrazole Hybrids of Electrophilic Natural Products as Promising Anticancer Agents

Alessia Da Fermo, Alessandra Bisi, Rebecca Orioli, Silvia Gobbi, and Federica Belluti *

Department of Pharmacy and Biotechnology, University of Bologna, Bologna, Italy

* Correspondence: Federica Belluti, Department of Pharmacy and Biotechnology, *Alma Mater Studiorum*-University of Bologna, Via Belmeloro, 6, 40126 Bologna, federica.belluti@unibo.it; Tel.: +39-0512099701

Content

Figure S1. Michael Addition reaction of a thiol-based moiety to the electron-rich fragment of chalcone or curcumin main templates.
Table S1. Docking Studies of the lead compounds described in the present review.
Table S2. The best-performing hybrid molecules are described in the present review.

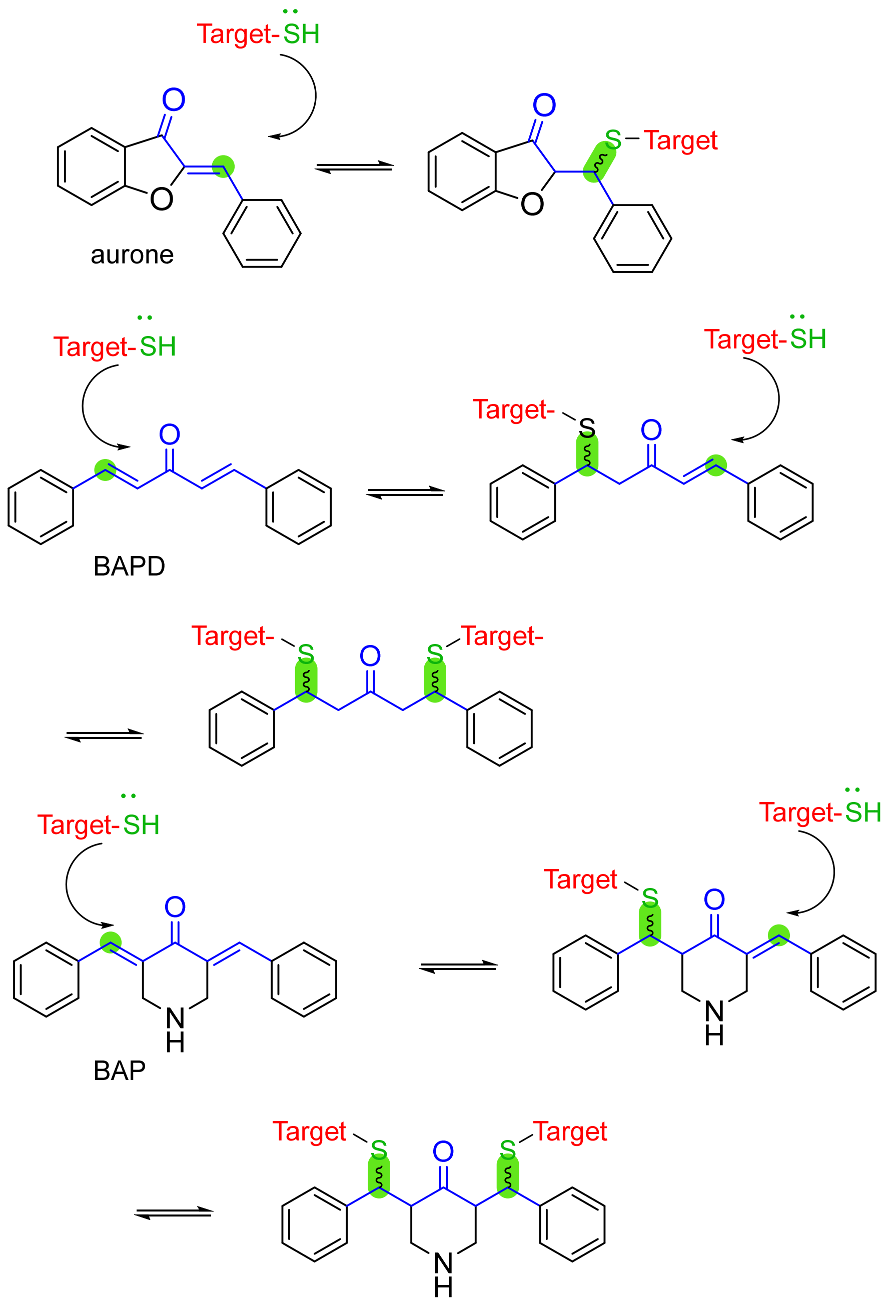

Figure S1. Michael Addition reaction of a thiol-based moiety to the electron-rich fragment of chalcone or curcumin main templates.

Table S1. Docking Studies of the lead compounds described in the present review. [a]See the main text for references

ID	Structure	Target		Ref [a]
**1**	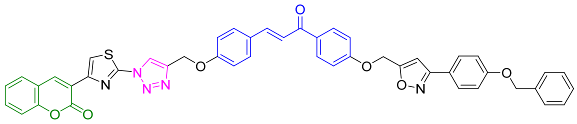	PDE-10A	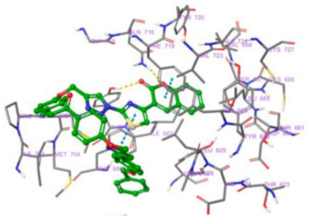	34
		EGFR	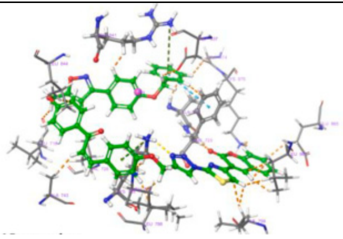	
**4a**	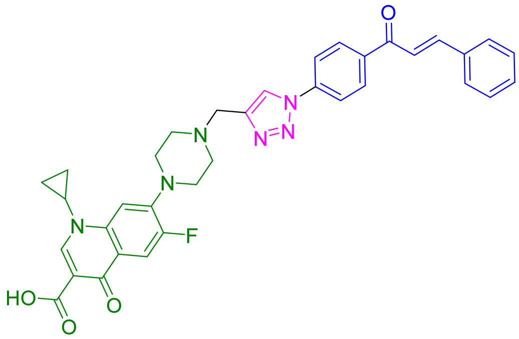	Topo I	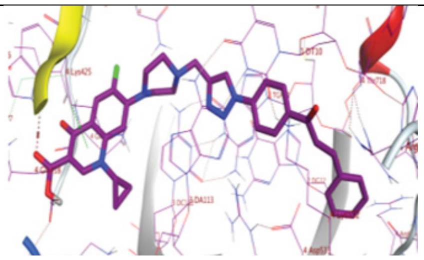	
		Topo II	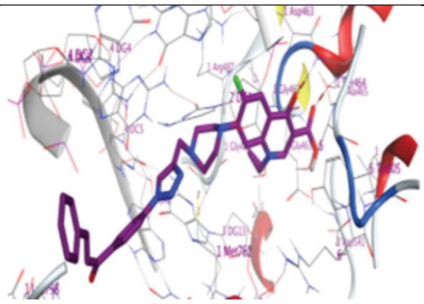	42
**4c**	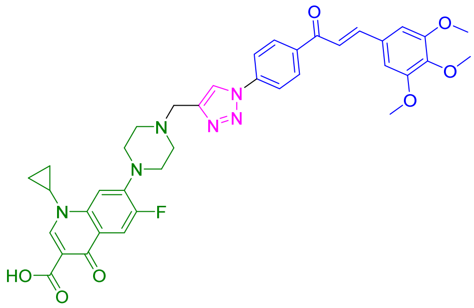	Topo II	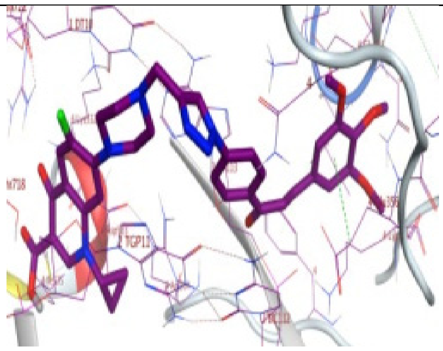	

<table>
<tr><td>7a</td><td></td><td>HDAC 8</td><td>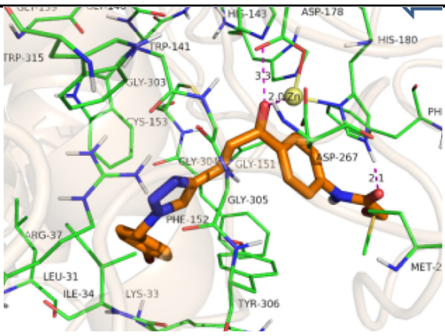
</td><td>47</td></tr>
<tr><td>10a</td><td></td><td>tubulin</td><td>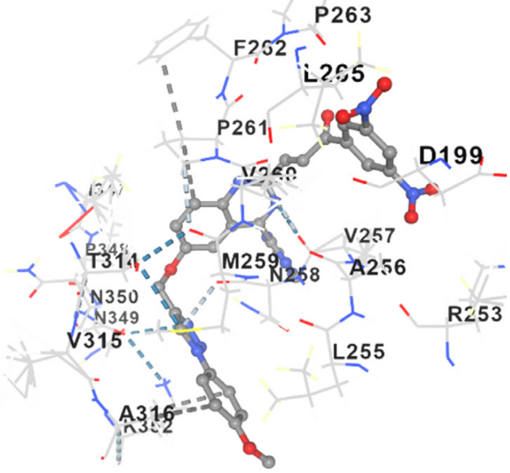
</td><td>53</td></tr>
<tr><td rowspan="2">11a</td><td rowspan="2"></td><td>VEGFR -2</td><td>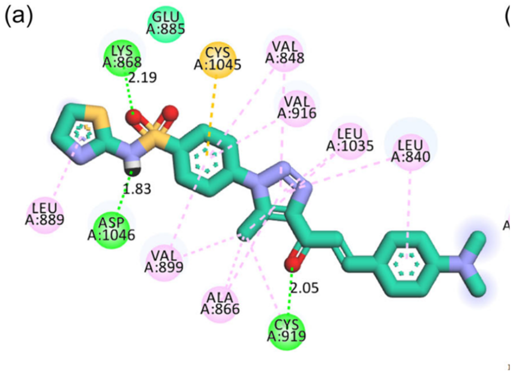
</td><td rowspan="2">55</td></tr>
<tr><td>EGFR</td><td>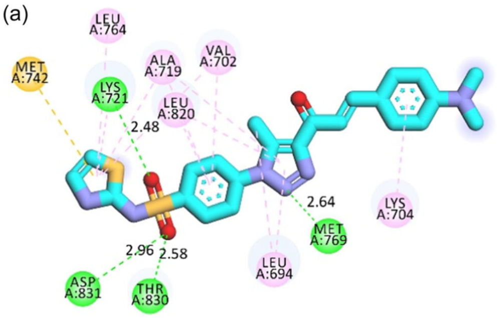
</td></tr>
</table>

**13a**	VEGFR-3	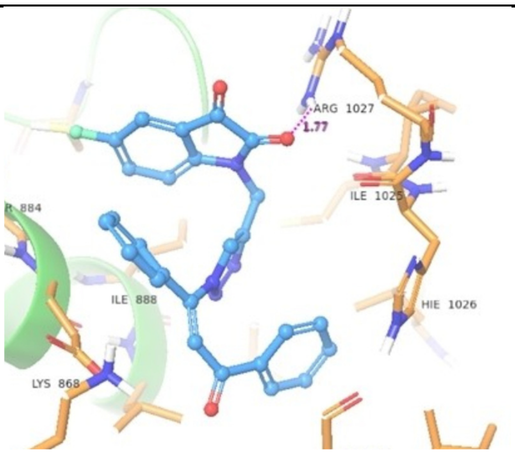	58
	EGFR	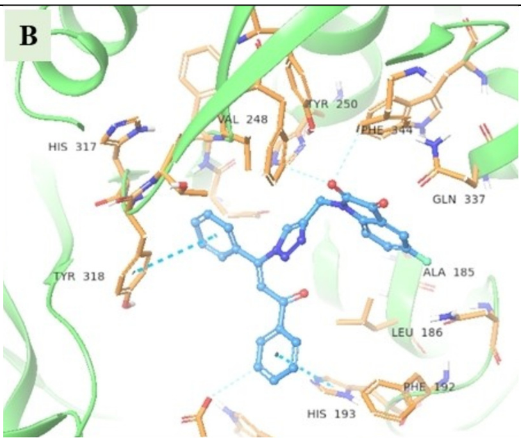	
**14a**	EGFR	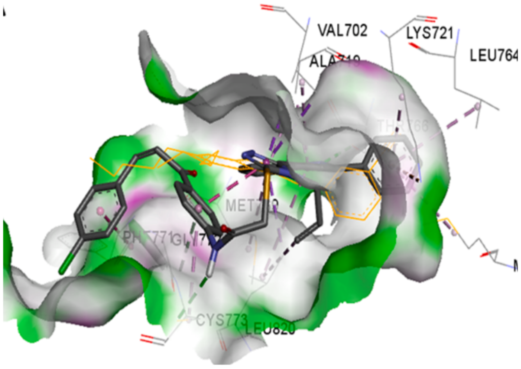	59
**16b**	EGFR	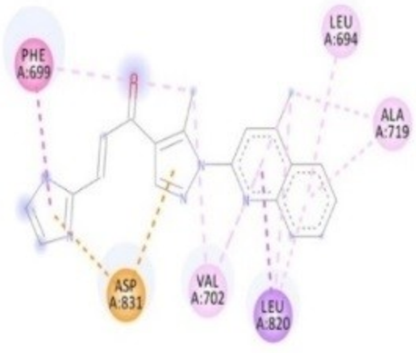	62

**17b**	EGFR	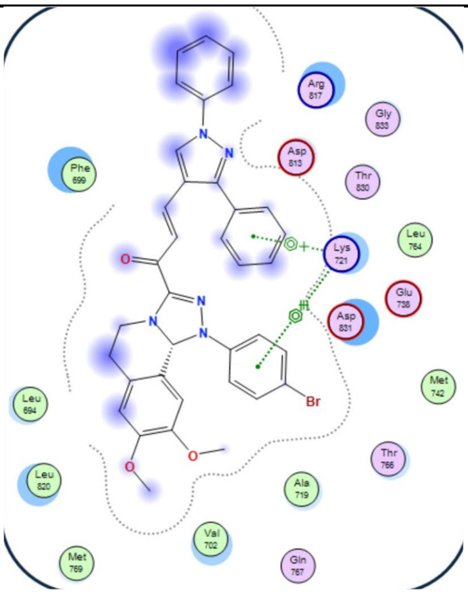	64
**20a**	APC-Asef protein	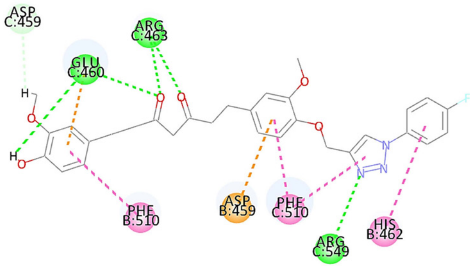	73
**25c**	HER2	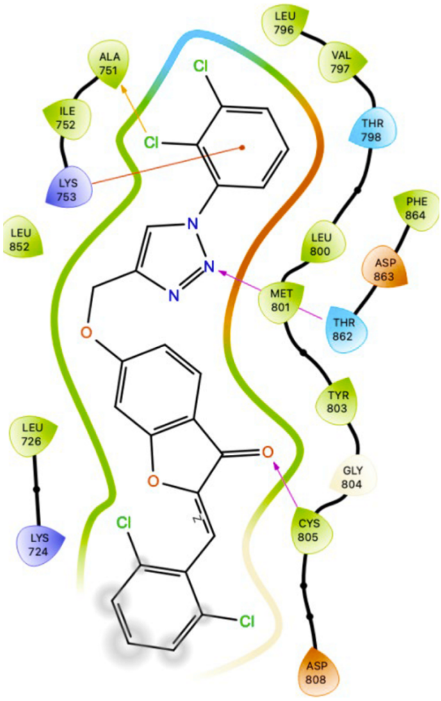	86

**28**	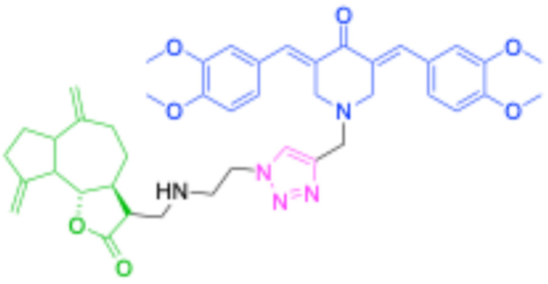	PKM2	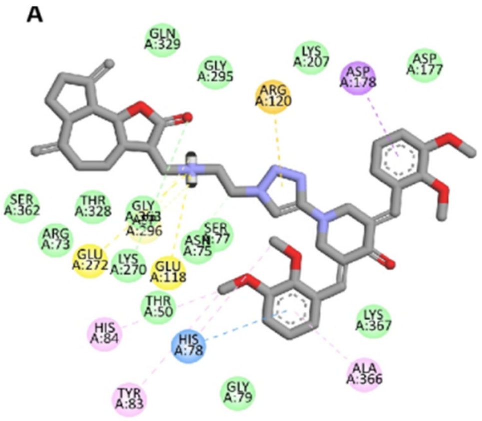A<br>GLN A:329<br>GLY A:295<br>LYS A:207<br>ASP A:178<br>ASP A:177<br>ARG A:120<br>SER A:362<br>THR A:328<br>ARG A:73<br>GLU A:272<br>LYS A:270<br>GLU A:118<br>SER A:77<br>THR A:50<br>HIS A:84<br>HIS A:78<br>LYS A:367<br>ALA A:366<br>TYR A:83<br>GLY A:79	91

Table S2. The best-performing hybrid molecules are described in the present review. [a]See the main text for references.

ID	structure	Citotiox IC_{50} (µM)	Target IC_{50}	REF[a]
**2b**	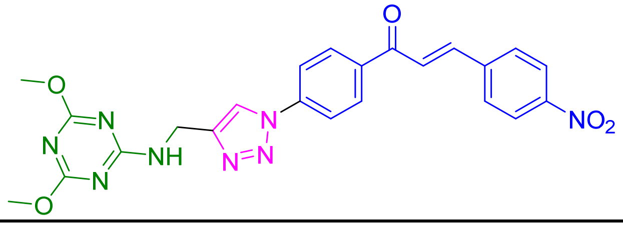	0.036 (HCT-116)	MMP-10 0.036 µM	38
**4a**	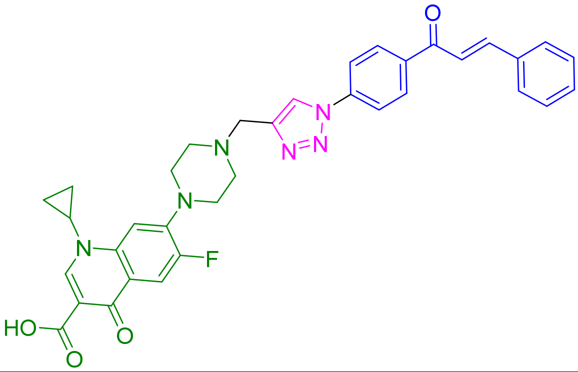	3.7 (HCT-116)	Topoisomerase I and II	42
**4c**	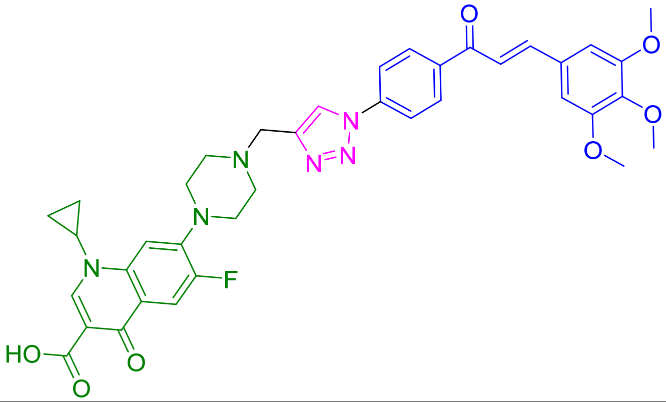	2.53 (HCT-116)	Topoisomerase I	
**5a**	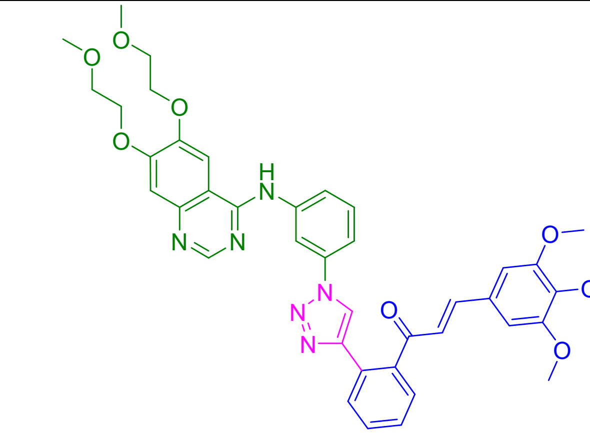	0.725 (SSC-25) 0.67 (Detroit 562)		43

**6c**	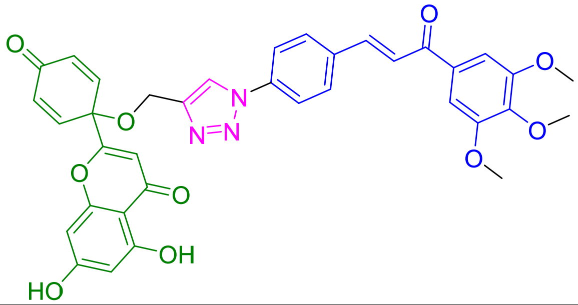	0.22 (MDA-MB-231) 0.30 (MCF-7)	45
**8a**	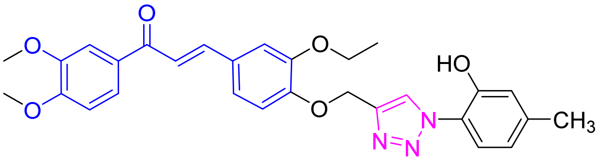	1.45 (MCF-7) 0.78 (MDA-MB-23)	48
**8b**	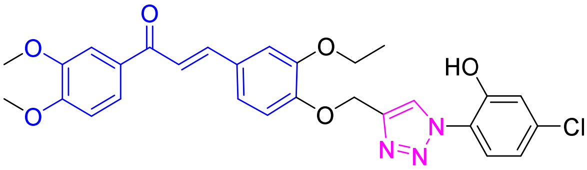	0.02 (MCF-7) 0.31 (MDA-MB-23	48
**10a**	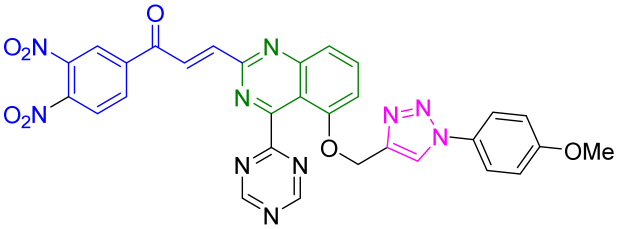	0.37 (PC3) 0.79 (A459) 0.13 (MCF-7)	53
**10b**	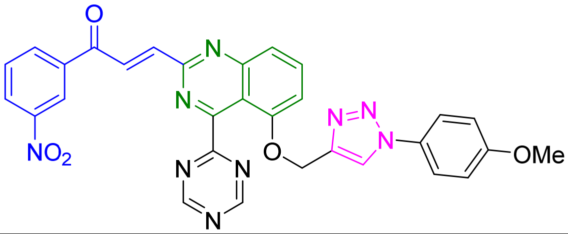	0.04 (PC3) 0.01 (A459) 0.09 (MCF-7)	
**18a**	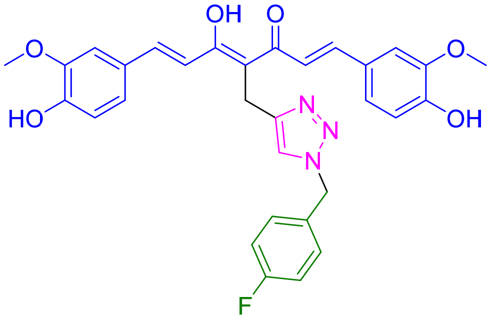	3.13 (CCRF-CEM)	71
**19a**	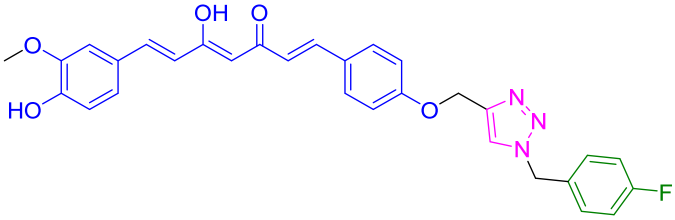	4.11 (HL-60 light irradiation) 6.25 MCF-7 light irradiation)	72

**20a**	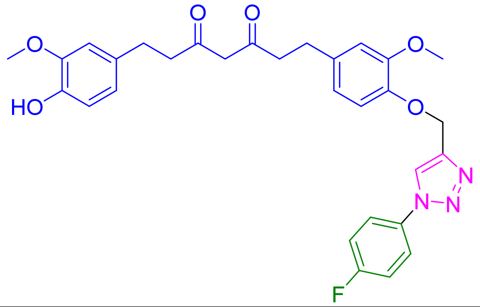	1.09 (HCT-116)		73
**20b**	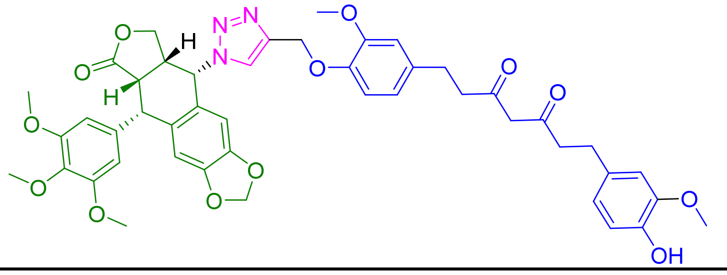	17.86 (HCT-116)		74
**21a-c**<br>**a:** D-mannose<br>**b:** D-lactose<br>**c:** D-maltose	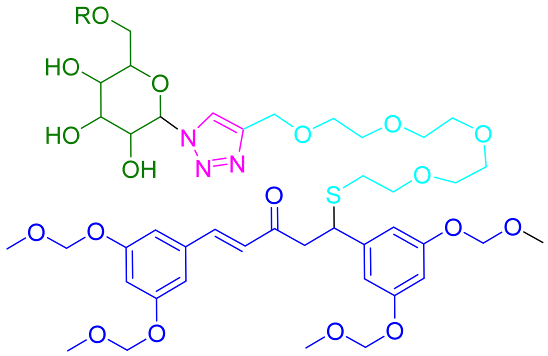	**a:** 0.44<br>**b** 0.43<br>**c:** 0.42<br>(HCT-116)		78
**9c**	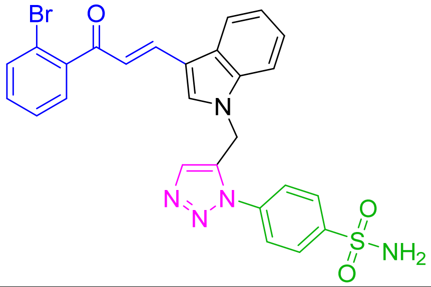	75.78 (Hcaix)	10 nM (CAXII)	52
**11a**	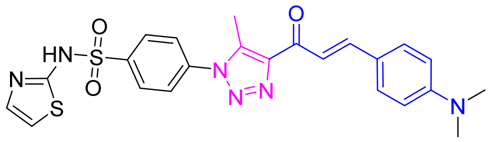	6.8 (MCF-7)	0.076 µM (EGFR)<br>0.085 µM (VEGFR)	55
**11b**	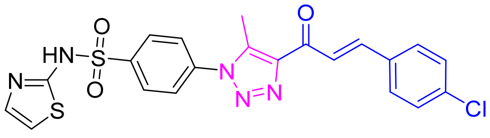	9.76 (MCF-7)	0.189 µM (EGFR)<br>0.108 µM (VEGFR)	
**13a**	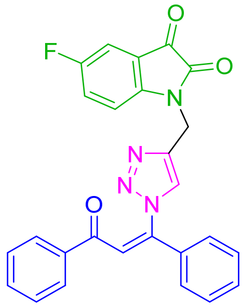	8.98 (HCT-116)<br>8.26 (OVCAR-10)	VEGFR2<br>EGFR	58

**14a**	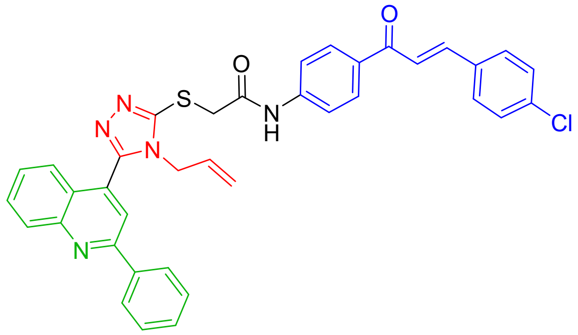	6.1 (A-549) 7.5 (MCF-7) 8.2 (Panc-1)	1.3 µM (EGFR) 3.8 µM (BRAF^{V600E})	59
**14b**	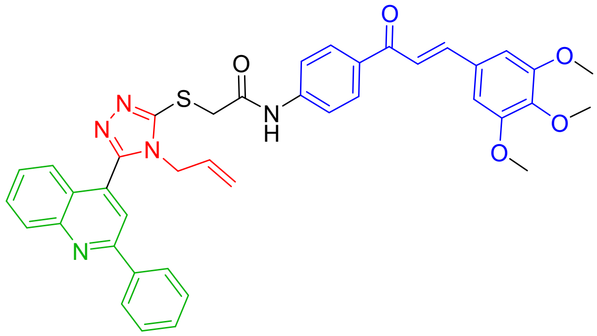	3.9 (A-549) 3.2 (MCF-7) 3.6 (Panc-1)	2.1 µM (EGFR) 1.6 µM (BRAF^{V600E})	
**16b**	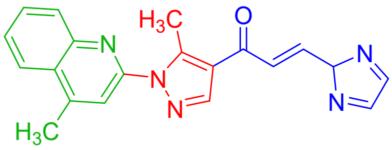	5.00 (PC-3) 2.14 (HCT-116)	EGFR	62
**17a**	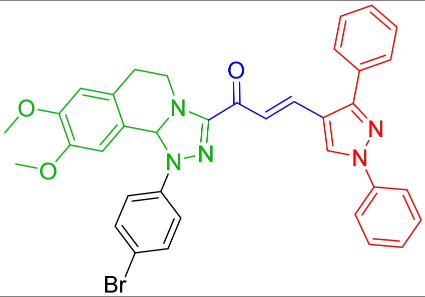	4.2 (HT.29) 2.3 (A-549) 4.21 (MCF-7)	0.031 µM (EGFR)	64
**17b**	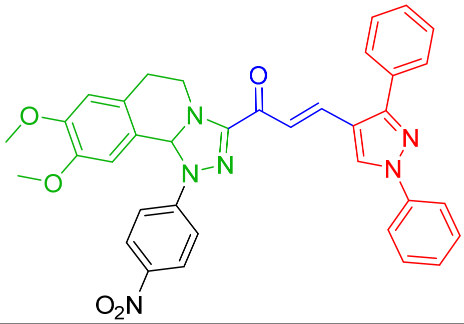	2.41 (HT.29) 1.15 (A-549) 5.53 (MCF-7)	0.023 µM (EGFR)	
**28**	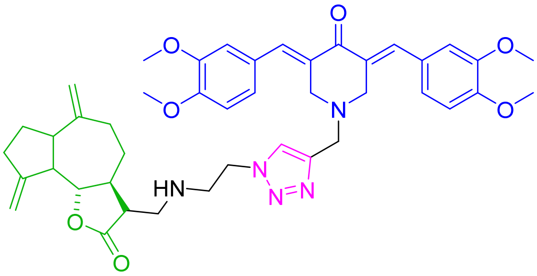	8.44 (MCF-7)		91
